# The impact of China’s value-added tax credit refunds on enterprise labor demand: Ex-ante analysis and ex-post test

**DOI:** 10.1371/journal.pone.0305249

**Published:** 2024-06-11

**Authors:** Yang Yang, Xin Yu, Jian Du

**Affiliations:** 1 School of Economics, Guizhou University of Finance and Economics, Guiyang, Guizhou, China; 2 School of Accounting, Guizhou University of Finance and Economics, Guiyang, Guizhou, China; Shandong University of Science and Technology, CHINA

## Abstract

This paper aims to explore the impact of China’s value-added tax (VAT) credit refunds policy on the enterprises’ labor demand through a paradigm combining ex-ante analysis and ex-post test. By introducing the VAT credit refunds into the production-decision model of the enterprise, calibrating the parameters and conducting the dynamic effects tests using the data of Chinese A-share listed enterprises, this paper finds that the labor employment of the pilot enterprises exhibits a *V-shaped fluctuation trend*. In the initial implementation of the policy, due to the existence of layoff costs, iso-cost line of the enterprise bends, which results in that the enterprise with a capital-labor substitution elasticity greater than 1 will not reduce labor hiring, as it has already deployed labor force before the implementation of the policy. When the enterprise enter the next production cycle where the labor force can be freely allocated, the labor employment of the enterprise with a capital-labor elasticity of substitution greater than 1 will decline compared to that without the policy. In the long run, as output increases, the labor demand will recover. The results of ex-post test are consistent with that of the ex-ante analysis. Additionally, heterogeneity test reveals that the greater the elasticity of capital-labor substitution of the sub-industry is, the more severe the degree of the *V-shaped fluctuation* is. Following the implementation of the policy, the continuous increase in enterprise output and capital stock verifies the relevant transmission mechanism. This study provides a more detailed perspective for comprehensively understanding the impact of VAT credit refunds policy on employment.

## 1. Introduction

Capital tax incentives (such as accelerated depreciation, value-added tax reform, etc.) usually reduce the price of capital used by enterprises [1–3], and how the decrease in capital price will affect the labor demand of enterprises has always been a great concern [4, 5].

Existing studies on this issue mainly focus on post-empirical tests. Specifically, for a certain policy, its impact on employment is verified ex-post through empirical methods such as difference-in-differences (DID) [6–15]. However, the theoretical analysis is ambiguous, and difficult to provide useful results, making it difficult to assess the policy effects in advance. At the same time, due to the lack of detailed ex-ante theoretical analysis, it is difficult to well explain the results of ex-post empirical testing. A relevant example is the value-added tax (VAT) credit refunds policy studied in this article.

In June 2018, the Ministry of Finance and the State Administration of Taxation of China issued the pilot policy of VAT credit refunds. It was clear that the final VAT credit of 18 major industries, such as special equipment manufacturing, research and experimental development, and power grid enterprises, would be refunded on the condition that the tax credit rating was A or B. The tax refund work was required to be completed before September 30, 2018. As a capital tax incentive policy, VAT credit refunds would inevitably affect the labor demand of enterprises. However, there are differences in the results of existing studies: One study [16] discovered that the VAT credit refunds effectively stimulated labor employment enterprises. But another [17] argued that the decline in capital prices resulting from this policy had a “crowding-out” effect on the scale of labor employment. The divergence may stem from differences in sample selection, with one study encompassing all industries except financial intermediation [16] and the other focusing solely on manufacturing [17]. However, careful analysis found that in addition to sample differences, there were also differences in the timing of policy implementation. If the impact of the policy is a non-monotonic pattern, choosing different periods could lead to different effects. Therefore, modeling the policy’s effects and conducting a dynamic analysis would be beneficial for accurately analyzing its impact. In other words, the best policy evaluation should be able to conduct a good pre-analysis and post-verification, and the results of the two can echo each other.

Taking the capital tax incentive policy of China’s VAT credit refunds implemented in June 2018 as the research object, the purpose of this paper is to establish a comprehensive analytical framework that includes both ex-ante analysis and ex-post test, aiming to provide a more detailed perspective for comprehensively understanding the impact of VAT credit refunds policy on employment. To this end, we try at first to establish a micro-mathematical model of VAT credit refunds affecting the labor demand of enterprises, which can be used for ex-ante assessment of the policy effects. We find that: At the beginning of policy implementation, due to the existence of layoff costs, the iso-cost line of enterprises bends, and enterprises with a capital-labor substitution elasticity greater than 1 and having completed labor configuration usually will not reduce labor hiring. When enterprises enter the next production cycle, where labor can be freely allocated and the output effect of the policy is not yet apparent, labor hiring in enterprises with a capital-labor substitution elasticity greater than 1 will decline compared to when the policy was not implemented. Subsequently, labor demand picks up as the output effect increases. The overall effect will be affected by the choice of period after the implementation of the policy. Furthermore, this paper conducts verification using the data of China’s A-share listed enterprises, the results of which are consistent with that of the theoretical analysis.

The potential contributions of this paper are twofold. Firstly, we introduce the VAT credit refunds into the production-decision model of enterprises, and calibrate the model parameters based on the data of Chinese enterprises. Based on this, we are able to make a pre-judgment on the dynamic impact of VAT credit refunds on the enterprises’ labor employment. This extends the theoretical analysis methods in this field. Secondly, we find that the impact of VAT credit refunds on employment is not monotonous “promotion” or “inhibition” as seen in existing studies, but rather exhibits a different degree of *V-shaped fluctuation trend* under different capital-labor substitution elasticities. We have verified this through theoretical analysis and empirical testing. Therefore, the evaluation of such policy effects should comprehensively consider the capital-labor substitution elasticity of the industry and the duration of policy implementation.

The rest of the paper is arranged as follows. Section 2 is the model analysis and ex-ante assessment of policy effects. Section 3 presents the empirical design, including model setting, data source, and variable description. Section 4 discusses the empirical results, including the main regression results and robustness test. Section 5 takes place the further test, including heterogeneity analysis and mechanism test. The last section presents conclusions and suggestions.

## 2. Mathematical model and ex-ante assessment

This section aims to analyze the impact of VAT credit refunds on labor employment from the perspective of a mathematical model. Firstly, the characteristics of the refund policy are analyzed. Then, the initial reaction of the pilot industry enterprises to the policy and the long-term effect of the policy are derived respectively. Finally, the dynamic effects of the policy are evaluated in advance with the calibration of relevant parameters.

### 2.1. Characteristics of the refunds policy

Value-added tax (VAT) is an indirect tax that is levied based on added value and collected in proportion. The taxable amount is equal to the output VAT minus input VAT. When the output VAT is not sufficient to offset the input VAT, a retained credit is formed. According to the research by Zhu [18], there are roughly seven reasons for the occurrence of retained credits. In addition to reasons such as the tax system, there are three common and difficult-to-avoid market reasons: (1) changes in the sales market, such as poor sales or price declines, resulting in sales revenue being less than the purchase amount; (2) concentrated purchasing amounts that are difficult to fully digest in the short term, such as seasonal purchasing; and (3) long-term infrastructure construction cycles and large investment amounts that have not yet resulted in sales revenue. In reality, it may be the result of the combined effects of multiple factors. It can be seen that the generation of the VAT credit is mainly due to the early construction of factories, the purchase of machinery and equipment, raw materials and other capital goods, but the sales of products have not yet been completed.

So, before the policy is implemented, enterprises should not expect that tax refunds will be taken into account when allocating factors. In other words, at the beginning of the implementation of the policy, the enterprise has completed the configuration of production factors. Therefore, the initial implementation and the regular implementation of policy should have different effects on enterprises, including initial response and long run effects.

**Fig 1:**
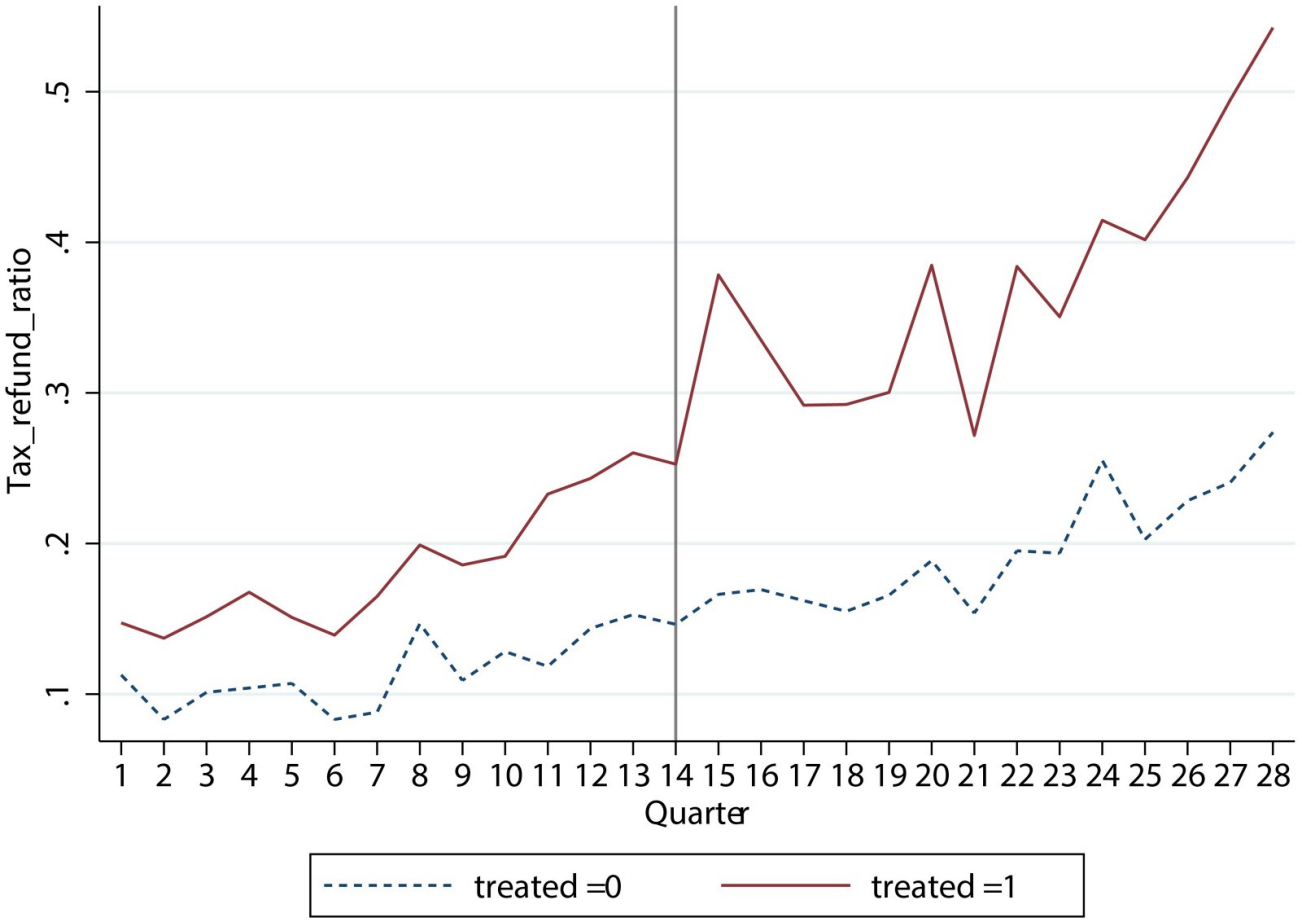
The enterprise average ratio of “refunds of taxes” to “2017 total assets” in pilot industries (solid line) and non-pilot industries (dashed line) from 2015Q1 to 2021Q4. It can be seen that there is a significant increase in 2018Q3 (the 15th quarter) and beyond for enterprises in pilot industries.

In terms of policy details, 2018 was the first year of large-scale implementation of VAT credit refunds, which was extended to the whole industry in 2019. And then, the policy was continuously implemented in subsequent years. Conditions of the 2018 VAT credit refunds are as follows: (1) The enterprise should be in the 18 pilot industries, or should be a power grid enterprise. (2) The tax credit rating of the enterprise should be A or B. The pilot policy was officially issued on June 27, 2018. And the refund work was required to be completed by September 30, 2018. In terms of accounting treatment, the received VAT credit refunds will be listed in the “refunds of taxes” item. Fig 1 shows the enterprises’ average ratio of “refunds of taxes” to “2017 total assets” in pilot industries (dashed line) and non-pilot industries (solid line) from 2015Q1 to 2021Q4. It can be seen that there is a significant increase in 2018Q3 (the 15th quarter) and beyond for enterprises in pilot industries.

The characteristics of the above VAT credit refunds serve as the foundation for theoretical analysis and empirical testing in the following paragraphs.

### 2.2. Production and taxation

To study the impact of VAT credit refunds on enterprise labor demand, we consider an economy with many heterogeneous enterprises. Referring to the research of Nie and Liu [19], it is assumed that enterprises have two production cycles. The first cycle is the period when the VAT credit refunds policy is just implemented, and the second cycle is the period when the policy continues to be implemented. Then the production of each cycle is divided into two stages. In Stage 0, which is a very short period, enterprises only invest without producing, and VAT credits will be formed at the end of this stage. In Stage 1, which is longer, enterprises sell all the output at the end of this period at a given market price. Assuming that enterprises only pay VAT. Before the implementation of the VAT credit refunds policy, the enterprises’ VAT credit will be carried forward to the next period for deduction (referred to as the carry-over deduction), and will be fully deducted after all the products are sold at the end of the Stage 1. When the policy is implemented, the enterprise applies for and receives the retention tax refunds at the end of Stage 0. We assume there is no delay in the model.

Suppose that an enterprise use capital and labor for production, the symbol *Y* represents output, *K* represents capital input, and *L* represents labor input. Its production function is in the form of constant elasticity of substitution (CES) production function proposed by Arrow et al. [20]:

$$Y_{c,1}=A{(\alpha K_{c,0}^{-\rho }+\beta L_{c,0}^{-\rho }\text{)}}^{-\frac{\mu }{\rho }}\;,\text{0}<\alpha ,\beta \leq 1;-1\leq \rho <\infty ;\mu >0$$ (1)

Where the subscript *c* represents the production cycle, taking the values 1 and 2. The number 0 at the right of the subscript represents Stage 0, 1 represents Stage 1, (*K*_*c*,0_, *L*_*c*,0_) represents the factor input combination at the Stage 0 of each cycle, *Y*_*c*_,_1_ represents the output at the end of Stage 1 of each cycle. *A* represents the total factor productivity, is exogenously given. *α* and *β* represent the allocation coefficients of capital and labor, *ρ* is the substitution coefficient, and *μ* is the parameter of returns to scale. The capital-labor elasticity of substitution *σ* can be obtained as follows [21]:

$$\sigma =\frac{1}{1+\rho }$$ (2)

When *ρ* = -1, *σ*→+∞, which is the production function of complete substitution between capital and labor. When *ρ* = 0, *σ* = 1, which is the Cobb-Douglas (C-D) production function, and the cross elasticity of capital price to labor is 0. When *ρ*→+∞, *σ*→0, which is the production function of complete complementarity between capital and labor. When *σ*>1, the inputs of capital and labor are gross substitutes. When *σ*<1, capital and labor inputs are total complements [22]. The main reason for choosing the CES production function in this paper is that the CES production function is more general and flexible than the C-D production function [23], and it can describe the production behavior of enterprises whose elasticity of substitution is not 1, which is crucial to the study of the substitution relationship between capital and labor.

It is further assumed that the market prices of capital and labor employed by the enterprise are given, which are $\bar{R}$ and $\bar{W}$ respectively. The enterprise can fire labor, but if it does so during the production cycle, it will incur a layoff cost $d*\bar{W}$(0<*d*<1) [24]. These costs may include invested training costs and economic compensation. The enterprise will not sell the capital (such as machinery, plant, etc.) it has just purchased in Stage 0, and it will pay VAT when purchasing capital, with the net amount that the output tax of Stage 1 minus the input tax of Stage 0. The enterprise has its self-owned initial capital *C*_0_ (*C*_0_>0), but due to financing constraints, the enterprise can only use the future-determined cash flow (VAT credit in this paper) as collateral for small-scale borrowing, and because of capital constraints, the enterprise has not yet reached the optimal production level without cost constraints. The market risk-free rate is *r*.

The assumption that an enterprise has constraints of self-owned funds is based on the following considerations: in practice, production with financial constraints is common [25–27], and it is generally difficult for enterprises to reach the level of profit maximization without cost constraints [28]. Therefore, it may be more in line with reality to analyze the impact of changes in capital price on labor demand under the framework of given cost constraints and maximizing output level.

### 2.3. Benchmark: When there is no refunds policy

This is the situation where the VAT credit refunds policy has not yet been implemented in the first production cycle of the enterprise, and the enterprise’s VAT credit will be carried forward for deduction, serving as a comparison for subsequent analysis. In Stage 0 of the production cycle, the enterprise hires labor, purchases capital, and pays VAT input tax on the purchased capital according to the tax rate *t*. Given the market price, the enterprise will choose the optimal amount of capital and labor input to maximize the output at the end of Stage 1, namely:

$$\begin{array}{ll} \underset{K_{1,0},L_{1,0}}{\text{max}}\;A{(\alpha K_{1,0}^{-\rho }+\beta L_{1,0}^{-\rho }\text{)}}^{-\frac{\mu }{\rho }} & \text{s.t}.\bar{W}L_{1,0}+(1+t)\bar{R}K_{1,0}-\frac{t\bar{R}K_{1,0}}{1+r}\leq C_{1,0} \end{array}$$ (3)

where, $t\bar{R}K_{1,0}/(1+r)$ is the income from small financing of the enterprise with future VAT credit as collateral. The *Lagrange* function is constructed and by finding the first-order conditions, the optimal capital and labor input is obtained:

$$\left\{\begin{array}{l} L_{1,0}^{*}=\frac{C_{1,0}}{\bar{W}+{\left[1+rt/(1+r)\right]}^{\frac{\rho }{\rho +1}}\bar{R}I}\\ K_{1,0}^{*}=\frac{I{\left[1+rt/(1+r)\right]}^{\frac{-1}{\rho +1}}C_{1,0}}{\bar{W}+{\left[1+rt/(1+r)\right]}^{\frac{\rho }{\rho +1}}\bar{R}I} \end{array}\right.$$ (4)

Where $I={\left(\alpha \bar{W}/\beta \bar{R}\right)}^{1/(\rho +1)}$. It can be concluded that the actual price of capital in Stage 0 is $[1+rt/(1+r)]\bar{R}$, and the actual iso-cost line of the enterprise is:

$$\bar{W}L_{1,0}+(1+\frac{rt}{1+r})\bar{R}K_{1,0}=C_{1,0}$$ (5)

The optimal configuration point $\left(K_{1,0}^{*},L_{1,0}^{*}\right)$ is the tangent point of the enterprise’s iso-output line and the actual iso-cost line.

### 2.4. Initial response to policy: Production Cycle 1

Before the implementation of the policy, the enterprise has no expectations, and in Stage 0, the enterprise has completed the configuration of capital and labor according to the carry-forward deduction, and the optimal configuration is $\left(K_{1,0}^{*},L_{1,0}^{*}\right)$, and VAT credit $t\bar{R}K_{1,0}^{*}$ is formed. Then, the refunds policy is implemented, and the enterprise applies for and gets the tax refunds at the end of Stage 0. After obtaining the funds, the enterprise can face two choices: (1) Putting the funds into bank, and getting the principal and interest to pay taxes at the end of the period. (2) Purchasing production factors and expanding output. It can be proved that the earnings of the enterprise from using the funds to expand production is greater than that from putting the funds into bank (See Appendix A in S1 File), so the enterprise will choose to expand production. For the enterprise that receives refunds, the cost constraint becomes:

$$\bar{W}L_{1,0}+(1-\frac{t}{1+r})\bar{R}K_{1,0}=C_{1,0}$$ (6)

This is a new iso-cost line, and it can be seen that compared with carry-forward deduction, the price of capital used by the enterprise receiving the VAT credit refunds decreases, from $[1+rt/(1+r)]\bar{R}$ falling to $[1\text{-}t/(1+r)]\bar{R}$.

With the new iso-cost line, an enterprise can redeploy capital and labor input in theory, and the optimal input combination $\left(K_{1,0}^{**},L_{1,0}^{**}\right)$ is (See Appendix B in S1 File):

$$\left\{\begin{array}{l} L_{1,0}^{**}=\frac{C_{1,0}}{\bar{W}+{\left[1-t/(1+r)\right]}^{\frac{\rho }{\rho +1}}\bar{R}I}\\ K_{1,0}^{**}=\frac{C_{0}I{\left[1-t/(1+r)\right]}^{\frac{-1}{\rho +1}}}{\bar{W}+{\left[1-t/(1+r)\right]}^{\frac{\rho }{\rho +1}}\bar{R}I}=\frac{C_{1,0}}{\bar{W}I^{-1}{\left[1-t/(1+r)\right]}^{\frac{1}{\rho +1}}+[1-t/(1+r)]\bar{R}} \end{array}\right.$$ (7)

Where $I={\left(\alpha \bar{W}/\beta \bar{R}\right)}^{1/(\rho +1)}$. It can be seen that if the enterprise is free to allocate capital and labor input, there will be a reduction in labor employment when -1<*ρ<*0.

However, due to adjustment costs, enterprises that have already allocated capital and labor inputs cannot freely allocate capital and labor by the new equal-cost line. First of all, enterprises generally do not reduce the capital (such as machinery, plant, etc.) that they have just purchased in Stage 0. Secondly, if an enterprise dismisses labor, it has to pay a firing cost, which is $d*\bar{W}$. In this way, in Production Cycle 1 when the VAT credit refunds policy is just implemented, the actual iso-cost line is a piecewise curve:

$$\left\{\begin{array}{ll} \bar{W}L_{1,0}\;+(1-\frac{t}{1+r})\bar{R}K_{1,0}=C_{1,0}\;, & L_{1,0}^{*}<L\leq \frac{C_{1,0}-(1-\frac{t}{1+r})\bar{R}K_{1,0}^{*}}{W}\\ \bar{W}L_{1,0}-d\bar{W}L_{1,0}+(1-\frac{t}{1+r})\bar{R}K_{1,0}=C_{1,0}-d\bar{W}L_{1,0}^{*}, & 0<L\leq L_{1,0}^{*} \end{array}\right.$$ (8)

**Fig 2:**
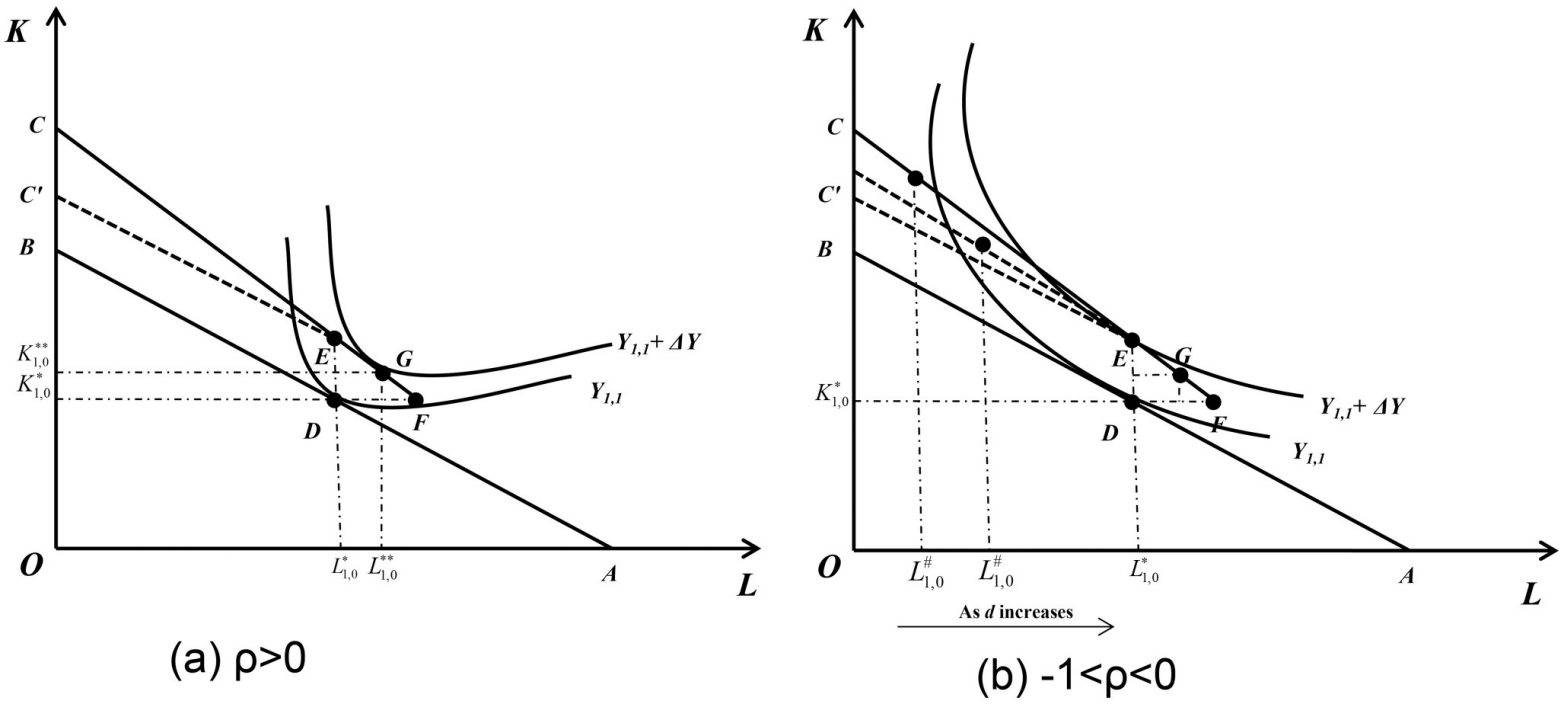
The reaction of different enterprises in the initial production cycle after the implementation of the VAT credit refunds policy. When *ρ*>0, which corresponds to the first equation in Eq (8), the enterprise can reach the new iso-cost line from the beginning configuration of $\left(K_{1,0}^{*},L_{1,0}^{*}\right)$ by hiring labor with all the tax refunds, or purchasing capital and hiring labor in a certain proportion. When -1<*ρ<*0, which corresponds to the second equation in Eq (8), the enterprise purchases capital with all the tax refunds from the beginning configuration of $\left(K_{1,0}^{*},L_{1,0}^{*}\right)$, and after reaching the new iso-cost line, it can increase capital by reducing labor, but due to paying layoff cost, iso-cost line bents, so that the capital saved by reducing labor cannot be obtained as much as that when the iso-cost line is not bent. The optimal labor input will increase with the increase of *d* when other conditions remain unchanged.

The first equation in Eq (8) corresponds to the case of *ρ*>0, as shown in Fig 2(a): The enterprise can reach the new iso-cost line from the beginning configuration of $\left(K_{1,0}^{*},L_{1,0}^{*}\right)$ by hiring labor with all the tax refunds, or purchasing capital and hiring labor in a certain proportion. That is, under the condition of *ρ*>0, enterprises that receive VAT credit refunds will increase their labor employment.

The second equation in Eq (8) corresponds to the case of -1<*ρ<*0, as shown in Fig 2(b): The enterprise purchases capital with all the tax refunds from the beginning configuration of $\left(K_{1,0}^{*},L_{1,0}^{*}\right)$, and after reaching the new iso-cost line, it can increase capital by reducing labor. But due to paying layoff cost, the enterprise’ iso-cost line bents, so that the funds saved by reducing labor cannot be used to acquire as much capital as when the iso-cost line is not bent. As shown in Fig 2(b), due to the existence of layoff costs, enterprises will not reduce labor employment at will. According to the iso-cost line of Eq (8), the optimal labor input when output is maximized can be obtained as follows:

$$L_{1,0}^{\text{\#}}=\frac{C_{1,0}-d\bar{W}L_{1,0}^{*}}{\bar{W}(1-d)+{\left[1-t/(1+r)\right]}^{\frac{\rho }{\rho +1}}\bar{R}I{\left(1-d\right)}^{\frac{1}{\rho +1}}}$$ (9)

After taking the partial derivative of *d*, it is found that the optimal labor input increases with the increase of *d* (the partial derivative is greater than 0) when other conditions remain unchanged, as shown in Fig 2(b). When *d* = 0, $L_{1,0}^{\#}=L_{1,0}^{**}<L_{1,0}^{*}$, and with the increase of *d*, $L_{1,0}^{\#}$ increases and gradually approaches $L_{1,0}^{*}$, then there must be a threshold *d*^#^, such that when *d = d*^#^, $L_{1,0}^{\text{\#}}=L_{1,0}^{*}$, and when *d>d*^#^, the optimal labor input will remain at $L_{1,0}^{*}$ and no longer change. Set $L_{1,0}^{\text{\#}}=L_{1,0}^{*}$, we can get:

$${\left(1-\frac{t}{1+r}\right)}^{\frac{\rho }{\rho +1}}{\left(1-d^{\text{\#}}\right)}^{\frac{1}{\rho +1}}={\left(1+\frac{tr}{1+r}\right)}^{\frac{\rho }{\rho +1}}$$ (10)

To obtain the approximate solution, the first-order Taylor formula will be expanded:

$${\left(1-d^{\text{\#}}\right)}^{\frac{1}{\rho +1}}=1-\frac{d^{\text{\#}}}{\rho +1}+ο(d)\approx 1-\frac{d^{\text{\#}}}{\rho +1}$$ (11)

then get:

$$d^{\text{\#}}=1-{\left(1+\frac{t+tr}{1+r-t}\right)}^{\rho }\approx -\rho \frac{t+tr}{1+r-t}$$ (12)

So, when -1<*ρ*<0, in the Production cycle 1 after the implementation of the policy, as long as *d* is not less than *d*^#^, the layoff cost will offset the decline in the capital price, so the enterprise will not reduce the employment of labor. What described so far leads to the following predictions:

**Implication1**: In the initial stage of the implementation of the VAT credit refunds policy, the enterprises received VAT credit refunds will increase labor employment in the case of *ρ>*0. But, as long as the firing cost is sufficiently large, that is, $d\geq d^{\#}\approx -\rho \frac{t+tr}{1+r-t}$, the enterprises received VAT credit refunds will not reduce labor employment in the case of -1<*ρ<*0.

### 2.5. Long-term effects of the policy: Production Cycle 2

From the above analysis, it can be seen that VAT credit refunds reduces the actual price of capital used by enterprises. In the long run, enterprises are no longer constrained by the “bent” iso-cost line and can freely allocate capital and labor within the new production cycle. With the labor price remaining unchanged, changes in the capital price will affect labor demand through both the “substitution effect” and “scale effect”. If the overall effect is substitution, the price of capital decreases and the demand for labor decreases. If the overall effect is complementary, the price of capital decreases and the demand for labor increases. The new optimal input combination $\left(K_{2,0}^{**},L_{2,0}^{**}\right)$ is:

$$\left\{\begin{array}{l} L_{2,0}^{**}=\frac{C_{2,0}}{\bar{W}+{\left[1-t/(1+r)\right]}^{\frac{\rho }{\rho +1}}\bar{R}I}\\ K_{2,0}^{**}=\frac{C_{2,0}}{\bar{W}I^{-1}{\left[1-t/(1+r)\right]}^{\frac{1}{\rho +1}}+[1-t/(1+r)]\bar{R}} \end{array}\right.$$ (13)

**Fig 3:**
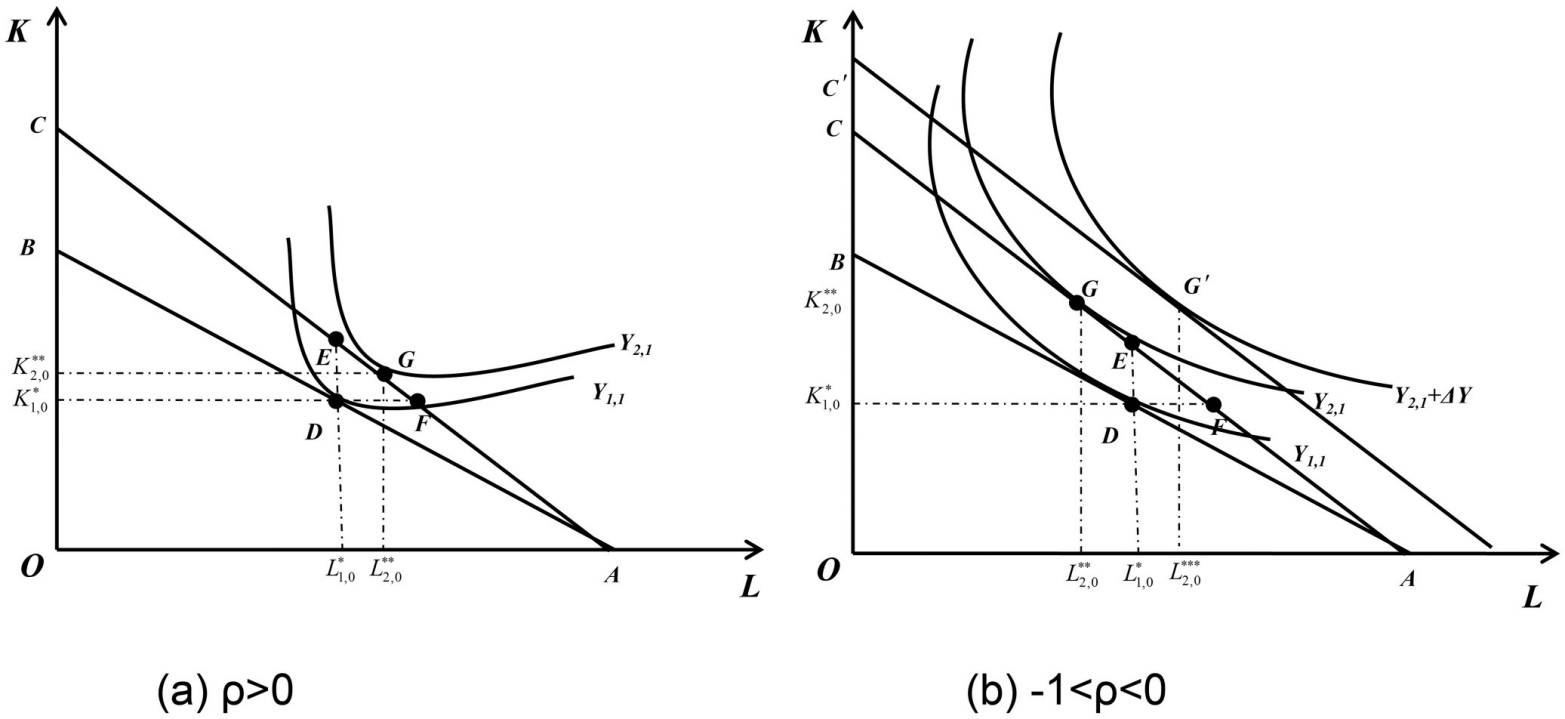
The long-term effect of the implementation of the VAT credit refunds policy on different enterprises. When *ρ*>0, the enterprises’ labor input will almost always increase. When -1<ρ<0, the enterprises’ labor input will decrease if the net profit of the enterprise in the Production Cycle 1 is 0, but will increase as net profit of the enterprise in the Production Cycle 1 is greater than the threshold.

We discuss it in two cases:

1. When *C*_2,0_ = *C*_1,0_. If the net profit of the enterprise in the Production Cycle 1 is 0, the enterprise’s own capital has not increased. In this case, the new production cycle (Production Cycle 2) is a repetition of the first production cycle, except that the enterprise can freely allocate capital and labor. For the optimal capital input, because the price of capital decreases, regardless of the value of *ρ* in the range of values, there is $K_{2,0}^{**}>K_{1,0}^{*}$. For the optimal labor input, it can be proved that: when *ρ*>0(*σ<*1), labor input increases, as shown in Fig 3(a). When *ρ* = -1(*σ*→+∞), capital and labor are completely substituted, which has no practical significance. When *ρ* = 0(*σ* = 1), the production function becomes Cobb-Douglas production function, and labor input remains unchanged. When -1<*ρ<*0(*σ>*1), at this time, labor input will decrease, as shown in Fig 3(b).
2. When *C*_2,0_ > *C*_1,0_. If the net profit of the enterprise in the Production cycle 1 is greater than 0, the capital of the enterprise will increase. At this point, when -1<*ρ<0*, *σ>*1, as investment increases, labor input is not necessarily less than $L_{2,0}^{**}$, as shown in point G′ in Fig 3(b), which means that $L_{2,0}^{**}\prime >L_{2,0}^{**}$ is possible. In other words, even if -1<*ρ<*0, *σ>*1, the VAT credit refunds policy does not necessarily reduce labor employment. By setting $L_{2,0}^{**}=L_{1,0}^{*}$, we can calculate the threshold that makes the labor force not decrease as follows:
$${C_{2,0}}^{\text{\#}}=\frac{\bar{W}+{\left[1-t/(1+r)\right]}^{\frac{\rho }{\rho +1}}\bar{R}I}{\bar{W}+{\left[1+tr/(1+r)\right]}^{\frac{\rho }{\rho +1}}\bar{R}I}C_{1,0}$$ (14)

At that time (*C*_2,0_ > *C*_2,0_^#^), the labor input of enterprises will also increase compared with that when the policy is not implemented. So the VAT credit refunds increases the output level of the enterprise and the net profit increases compared with the carry-forward tax deduction. As long as the net profit at the end of the first period is not negative, there is *C*_2,0_ > *C*_1,0_. Thus, when *ρ*>0, *C*_2,0_ > *C*_2,0_^#^is satisfied. Therefore, it can have the following implication:

**Implication 2**: The long-term effect of the VAT credit refunds policy on the labor demand of enterprise depends on the sign of *ρ* and the degree of increase of self-own capital *C*. When *ρ*>0, labor employment will increase compared to when the policy is not implemented. When *ρ* = 0 or *ρ* = −1, labor employment will remain unchanged compared to when the policy is not implemented. When -1<*ρ*<0, labor employment will decrease, but the increase in labor demand caused by the increase in output level can offset some of the decrease. When *C*_2,0_ > *C*_2,0_^#^, the enterprise’s labor input will increase compared to when the policy was not implemented.

### 2.6. Parameters calibration and ex-ante analysis

According to the above analysis, the price of capital used by enterprises receiving the VAT credit refunds decreases, compared with that of VAT credit carry-forward deduction to the next period. As capital price declines, the response of the enterprise is divided into initial response (the first production cycle) and long-term effects (the second production cycle). The demand for labor varies from period to period and depends on the parameters *ρ*(*σ*), *μ*, *t*, *r*, and the main parameters are *ρ* or *σ*, that is, the elasticity of substitution of capital and labor.

Generally, we suppose the production cycle of an enterprise is to be 1 year. As for *μ*, according to the research of Deng and Li [29], the return to scale of Chinese industrial enterprises is stable at around 1.2. As for *t*, after May 1, 2018, China’s VAT rate is 16%. For *r*, the average return rate of bank wealth management in 2018 is about 4%. As for the dismissal cost, according to the Labor Contract Law, the enterprise should pay one month’s salary before the end of one year, so *d* = 1/12 = 0.083 is set here. For *σ*, according to the capital-labor elasticity of substitution of various industries estimated by Chen and Chen [30], the value ranges from 1.31 to 3.98, with an average of around 2, and it is more certain that the elasticity of capital-labor substitution in all industries of China is greater than 1 [31], that is, -1<*ρ*<0.

So, after the calibration of various parameters of Chinese enterprises, it can be concluded that:

1. When *t* = 16% and *r* = 4%, the actual price of capital used by enterprises decreases from 1.0061 to 0.8461.
2. Initial effects. When *σ* = 2, *ρ* = -0.5, *t* = 16%, *r* = 4%, we get d^#^≈0.074; when *σ* = 1.31, *ρ* = -0.237, *t* = 16%, *r* = 4%, we get d^#^≈0.035; when *σ* = 3.98, *ρ* = -0.749, *t* = 16%, *r* = 4%, we get d^#^≈0.111. However, the actual *d* is above 0.083, so in the initial stage of policy implementation (the first cycle), most enterprises usually will not terminate employees.
3. Long-term effects: Based on data from China’s A-share listed companies from 2013 to 2021, the actual calculated average capital return rate is 1.41%, and 90% of the enterprises have a profit margin of less than 16.45%. Therefore, the average return on capital of listed companies is difficult to reach 18%. Thus, at the beginning of the long-run effect (say, the second year), the scale effect of the fall in the price of capital usually does not outweigh the substitution effect, and labor employment is reduced sharply.

Based on the above analyses, this paper draws the following inferences:

**Inference 1:** In the initial stage of the implementation of the VAT credit refunds policy, the average number of labors employed by enterprises will not decrease.
**Inference 2:** In the subsequent stage of the implementation of the VAT credit refunds policy, the average number of labors employed by enterprises will decrease, and then recover with the increase of output.

## 3. Design of the empirical test

### 3.1. Strategy

Existing literature in the empirical analysis of the effect of the VAT credit refunds policy mainly adopts the classic DID method. Taking 2018 as the dividing point between before and after the policy implementation, it treats the 18 pilot industries as the treatment group and other non-pilot industries as the control group [16, 17, 32, 33]. However, we believe that after the implementation of the VAT credit refunds policy in 18 industries and power grid companies in 2018, it was extended to all industries in 2019 and afterwards. In other words, there is no strictly defined control group based on industry segmentation after 2019. It can also be seen from the trend of tax and fee returns in non-pilot industries from 2018 (see Fig 1) that the proportion of tax and fee returns in total assets has increased significantly in the fourth quarter of 2019 and beyond, and the trend is consistent with that of the experimental group in 2018. This fully indicates that if we continue to group by whether it is a pilot industry in 2018, it will necessarily contaminate the treatment effect after 2019.

**Fig 4:**
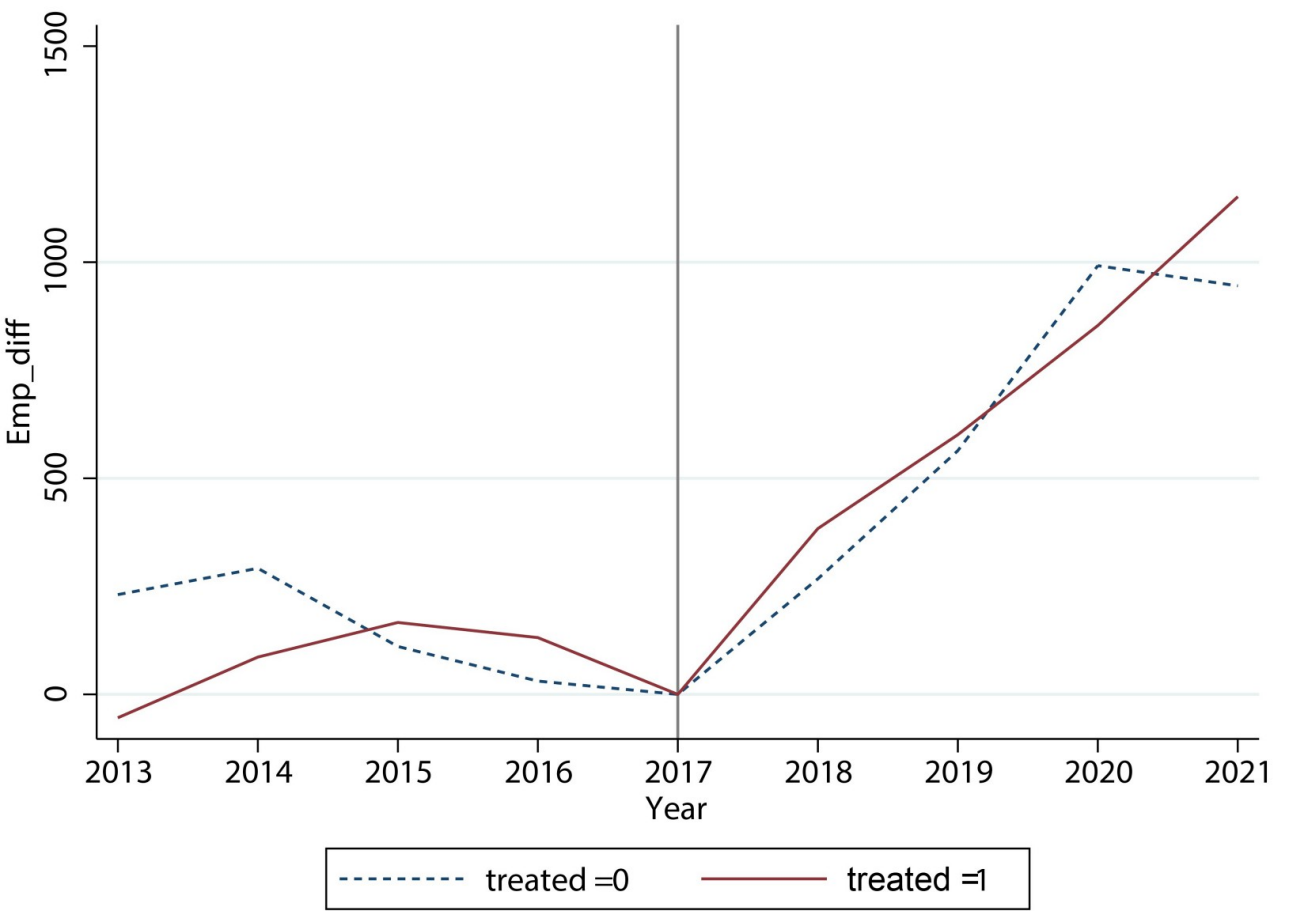
A comparison of enterprises’ employment between pilot and non-pilot industries. There was no significant parallel trend in employment before 2018, and both groups saw a significant increase in employment numbers and maintained almost the same trend after 2018. This figure was generated in STATA17.

Moreover, we took the number of employees at the end of 2017 as the base to calculate the average difference between the number of employees at the end of each year and that at the end of 2017. As shown in Fig 4, a comparison of enterprises’ employment between pilot and non-pilot industries, shows that there was no significant parallel trend in employment before 2018, and both groups saw a significant increase in employment numbers and maintained almost the same trend after 2018. Therefore, non-pilot industries are not suitable as a control group for DID. Additionally, although there were 18 pilot industries for value-added tax credit refunds in 2018, not all enterprises were eligible to apply (there were differences in taxable levels and credit balances among enterprises), and even if they obtained credit refunds, there were differences in the amount of refunds, which may make the policy effects of the VAT credit refunds “drowned” in the average performance of enterprises within the pilot industries, making it difficult to discern. Based on these three reasons, we believe that the conventional DID method cannot effectively evaluate the effect of the VAT tax credit refunds policy in 2018, and other methods need to be explored.

In this paper, through theoretical analysis, we know that the VAT credit refunds policy effects can be roughly divided into initial effect, medium-term effect and long-term effect, and the results in different periods are different. Due to examine this dynamic effects of VAT credit refunds on employment, we draw on the research of Curtis et al. [34] and Callaway et al. [35] to use the event study method with continuous treatment variable. Taking listed enterprises from all 18 pilot industries in 2018 as the research sample, with only a treatment group and no control group, we construct an intensity indicator that reflects the strength of VAT credit refunds.

However, how to construct this intensity indicator is the most difficult part of this article. If we know the specific amount of VAT credit refunds received by the enterprise in the third quarter of 2018, we can take the ratio of this amount to the total assets of 2017 as the intensity of the tax refunds. But, due to the confidentiality of tax data, we cannot obtain the specific amount of VAT credit refunds received by these companies publicly. We only manually collected this data from less than 30 companies. Therefore, we need to find a proxy variable.

Referring to the research of Wu et al. [33], we take “refunds of taxes” by enterprises as a breakthrough point for addressing this challenge. The VAT credit refunds policy of 2018 was officially announced on June 27, 2018, with a requirement to be completed by the end of September 2018. As a result, enterprises’ VAT credit refunds were concentrated in the third quarter of 2018. Furthermore, it is known that in terms of accounting treatment, enterprises record the VAT credit refunds received under the “refunds of taxes” account, which is an immediate effect at the end of the third quarter of 2018 with no lag. Therefore, changes in the “refunds of taxes” account in the third quarter of 2018 can serve as an estimate of the VAT credit refunds. Wu et al. found that the explanatory power of the increase in “refunds of taxes” as a single variable for the actual VAT credit refunds received by listed enterprises is at least 70.0%.

To reflect the intensity of the VAT credit refunds rather than the absolute values, we divide it by the enterprise’s total assets in 2017. Then, we use the data before the policy implementation and fit the value for the third quarter of 2018 through the OLS method, assuming that the policy had not been implemented. By subtracting the fitted value assuming no policy implementation from the actual value, we can obtain an estimated value for the intensity of the enterprises’ VAT credit refunds. The specific steps for calculating the intensity of the VAT credit refunds are as follows:

*Step1*. Screen the “refunds of taxes” data of listed enterprises in the pilot industries for the third quarter of 2018, and divide it by the total assets at the end of 2017 to obtain the actual tax refunds ratio (*Tax_refunds_R*) for each enterprise.
*Step2*. Select the “refunds of taxes” values of listed enterprises in the pilot industries for the 6 quarters prior to the second quarter of 2018, and divide them by the total assets at the end of 2017 respectively. Perform OLS regression on the obtained ratios by quarter to obtain the fitted values of “refunds of taxes” in the third quarter of 2018 (*Tax_refunds_E*). The fitting outcomes may be related to the selected time span. Therefore, in the later section of the article, we use a time span of 10 quarters as a robustness test.
*Step3*. Calculate the intensity of the VAT credit refunds, *Tax_refunds = Tax_refunds_R—Tax_refunds_E*.

Because the implementation time of the VAT credit refunds is relatively concentrated (in the third quarter of 2018) and the accounting treatment is straightforward, our estimated value should be relatively close to the true intensity of the VAT credit refunds. We use two methods to test the validity of proxy variables.

First, difference test between groups. The above *Step1-Step3* calculates the difference between the actual value and the fitted value of the tax refunds ratio in the third quarter of 2018, serving as a proxy variable for the intensity of the enterprises’ VAT credit refunds. As shown in Table 1, during the third quarter of 2018 (2018Q3) when the policy of VAT credit refunds was implemented, the mean difference between the actual value and the fitted value of the tax refunds ratio is significant (at a significance level of 1%), indicating that the implementation of the policy significantly increased the tax refunds ratio of enterprises. If we use the same method of *Step1-Step3* to move backward by one period, that is, using the data of the 6 periods before the second quarter of 2018 to fit the tax refunds ratio value of the second quarter of 2018 and compare it with the actual value. As shown in Table 1, in the second quarter of 2018 (2018Q2), the mean difference between the actual value and the fitted value of the tax refunds ratio by the same method is not significant. Similarly, we also calculated the mean differences between the actual value and the fitted value of the tax refunds ratio in the first quarter of 2018 (2018Q1) and the fourth quarter of 2017 (2017Q4), and the differences between the two are also not significant. Therefore, the significant mean difference between the actual value and the fitted value of the tax refunds ratio in the third quarter of 2018 captures the impact of the policy of VAT credit refunds on the tax refunds ratio of enterprises.

**Table 1:** Difference test between groups.

Time	Group	Observations	Mean	t value	Difference (Actual Value -Fitting value)
2018Q3	Fitted value (*Tax_refunds_E*)	1785	0.252403	5.247	0.087982***
	Actual Value (*Tax_refunds_R*)	1785	0.34039		
2018Q2	Fitted value (*Tax_refunds_E*)	1576	0.240811	-1.201	-0.017522
	Actual Value (*Tax_refunds_R*)	1576	0.223290		
2018Q1	Fitted value (*Tax_refunds_E*)	1598	0.239139	-0.826	-0.012202
	Actual Value (*Tax_refunds_R*)	1598	0.226937		
2017Q4	Fitting value (*Tax_refunds_E*)	1432	0.232301	0.769	0.011897
	Actual Value (*Tax_refunds_R*)	1432	0.244197		

Significance levels:

*** *p* < 0.01,

** *p* < 0.05,

* *p* < 0.1

*Source*: China Economic and Financial Research Database (CSMAR)

Second, comparison based on the partially available sample data. Although we cannot obtain the VAT credit refunds data for all enterprises, we can obtain real data for a very small subset of them. These data can be used to test the accuracy of our calculated values. Table 2 presents the comparison between our calculated values and the actual values.

**Table 2:** Measurement error of the intensity of VAT credit refunds.

No.	Stock code	Calculated intensity of VAT credit refunds	Real intensity of VAT credit refunds	Measurement error
1	000413	0.595683	0.360659	0.651653
2	000559	0.240516	0.150015	0.603275
3	000957	1.295342	1.340082	-0.033386
4	002132	0.510556	0.509748	0.001586
5	002190	1.039259	0.994216	0.045305
6	002407	1.351272	1.361090	-0.007214
7	002491	0.879413	0.935845	-0.060301
8	002855	0.959574	1.022875	-0.061886
9	002921	0.375839	0.732652	-0.487016
10	300098	0.859191	0.987176	-0.129647
11	300450	0.445928	0.678061	-0.342348
12	300642	0.124690	0.282998	-0.559397
13	600085	0.038337	0.167231	-0.770756
14	600096	0.452200	0.304384	0.485625
15	600470	0.486606	0.535136	-0.090688
16	600691	0.705397	0.806879	-0.125771
17	600839	0.063204	0.054460	0.160573
18	601208	0.243256	0.271333	-0.103477
19	603186	1.199926	1.106665	0.084272
20	603663	0.499956	0.452976	0.103714

*Source*: Manually collated and calculated based on the annual reports of listed companies.

From Table 2, it can be seen that based on the manually collected data, the average absolute error between the estimated VAT credit refunds intensity and the true values is 24.54%. Compared to the conclusion of Wu et al. [33] who used 20 listed companies to achieve an explanatory power of over 70%, our results (with explanatory power of 75.56%) are similar.

### 3.2. Model and variables

As mentioned in the empirical strategy, we adopt the event study method for the analysis in order to better demonstrate the dynamic effects of the policy. The event study method can be traced back to Dolley’s research on the impact of stock splits on stock prices in 1933 [36]. In recent years, the event study method has been used as a standard step for the parallel trend test of DID analysis [37]. In fact, the event study method can be used as an independent econometric method and is more suitable for evaluating the dynamic effects of policies. Generally speaking, the event study method does not need to distinguish between the treated group and the control group, and it can be tested using different policy intensities among individuals [37], for example, the research of Nunn and Qian in 2011 [38]. Certainly, dividing the research subjects into an treated group and a control group is also a case of the event study method. In this paper, we only use the data of 18 pilot industries, so there is no control group. We test the employment effects of the policy of VAT credit refunds based on the different policy intensities among enterprises. The model is as follows:

$$\text{ln}\;Emp_{i,t}=\alpha +\sum_{k=2015,k\neq 2017,}^{2021}\beta_{k}\left[Tax\_refunds_{i}\times {Ι}_{k}(k=t)\right]\;+\gamma X_{i,t-1}+u_{i}+\lambda_{t}+\varepsilon_{it}$$ (15)

Where ln*Emp*_*i*,*t*_ represents the explained variable for enterprise *i* in year *t*. *Tax_refunds* represents the intensity of VAT credit refunds received by enterprises. I*k*(*k* = *t*) is an indicator function, when *k = t* equals 1, otherwise 0. The coefficients *β*_201_ through *β*_202_ describe the relative outcome changes for enterprises that benefit most from VAT credit refunds relative to 2017. *u* and *λ* represent individual fixed effect and time fixed effect respectively. *ε* is the random disturbance term. *X* is the set of control variables. Variables are described in detail as follows:

1. Explained variable (ln*Emp*). Drawing on the existing studies [9, 39–42], the natural logarithm of the number of employees at the end of the year is used to represent the scale of labor demand.
2. Core explanatory variable (cross-product of *Tax_refunds* and I_*k*_(*k* = *t*)). In the previous text, we have already described the calculation method for *Tax_refunds*. Obviously, the greater *Tax_refunds* is, the greater the effect will be on it. I_*k*_(*k* = *t*) is used as the dummy variable of time, so as to compare the employment effects of enterprises with different *Tax_refunds* at the same time point.
3. Control variable (*X*). With reference to existing literature [11, 43–45], the control variables selected in this paper include:

Enterprise Size (*Size*), which is equal to the natural logarithm of total assets at the end of the year. The larger the enterprise’s size, the larger the scale of labor employment.

Return on assets (*ROA*), which is equal to the ratio of the net profit of the current year to the total assets at the end of the year. The stronger the profitability of the enterprise, the smaller the capital constraint, and the size of the labor force will expand.

Asset-liability ratio (*Lev*), which is equal to the ratio of total liabilities to total assets at the end of the year. A company with high debt may reduce the size of the labor force to reduce costs.

Capital intensity (*Fix*), which is equal to the ratio of the net fixed assets to the total assets at the end of the year. When the total asset size is fixed, the greater the capital intensity, on the one hand, indicating that there are more machines and equipment and more labor force needed to be employed; but on the other hand, due to the substitution relationship between capital and labor, the employment of labor force may also be less.

Enterprise growth(*Growth*), which is measured by the annual growth rate of the company’s operating revenue. The faster the growth rate of operating revenue, the better the growth of the company and the more labor force it will employ.

Ownership concentration (*Top1*), which is measured by the shareholding ratio of the largest shareholder. Enterprises with high equity concentration have relatively faster labor employment decisions.

The age of the enterprise (*Age*), which is equal to the natural logarithm of the difference between the data year and the year of establishment of the enterprise. Enterprises with a long establishment time may have greater labor demand than those with a short establishment time.

The per capita salary (*Wage*), which is equal to the cash paid to and for employees in the current period divided by the number of employees and the natural logarithm is taken. Salary levels will affect the employment scale of enterprises, and higher salary levels will attract more labor force, but excessively high salary levels will also inhibit the employment capacity of enterprises.

Industry concentration (*HHI*), which is measured by the Herfindahl-Hirschman index. In this paper, the share of total assets of enterprises in the total assets of all enterprises in the industry (two-digit industry code) is used to calculate the industry concentration index. The larger the share held by an enterprise (high industry concentration), the greater the number of labor force absorbed.

The level of economic development (*PGDP*), which is measured by the natural logarithm of GDP per capita of the province. Areas with a high level of economic development tend to have a larger population and a larger scale of employment by enterprises.

The central role of the control variable is to ensure that the conditional independence hypothesis holds [46], which means that the policy processing variable, that is, the explanatory variable, is not related to the random disturbance term *ε*. To alleviate the problem of possible endogeneity or “bad” control variables [47], this paper learns from the practice of Li et al. [48] and Yu et al. [41], and all control variables are treated with a lag of 1 period. At the same time, this paper also controls the individual and time fixed effects.

### 3.3. Data

Given the availability of micro-enterprise data, this paper selects the annual data of China’s A-share non-financial listed companies as research samples. The source of all company data is the China Economic and Financial Research Database (CSMAR), and that of regional economic data is the China Statistical Yearbook.

The target policy of this paper is the VAT credit refunds policy implemented in 2018. The period we selected is from three years before the policy implementation to four years after, which is from 2015 to 2021. In the data processing, this paper further eliminates abnormal enterprises such as ST, *ST, or delisting enterprises [49, 50]. ST stocks refer to enterprises that have suffered losses for two consecutive years and are subject to special treatment. *ST stocks refer to enterprises that have suffered losses for three consecutive years and are at risk of being delisted. And, because not all enterprises in the pilot industries will receive VAT credit refunds, only samples with *Tax_refunds* > 0 will be retained.

To eliminate the large abnormal changes in the number of employees caused by abnormal values such as statistical errors, we eliminate the data with less than 200 employees (this screening step is consistent with Wang and Ni [11], Zeng and Chen [45]), and the samples with standardized values at quantiles below 1% are winsorized. Then, companies that were listed in 2018 and later are also deleted. Finally, 5184 observed values are obtained, involving 945 enterprises. The descriptive statistical results of the main variables are shown in Table 3.

**Table 3:** Descriptive statistical results of the main variables.

Variable	Observations	Mean	SD	Min	Max	p25	p50	p75
*Emp*	5814	7.843	1.139	5.303	12.57	7.031	7.736	8.549
*Tax_refunds*	5814	0.263	0.431	0	3.774	0.0304	0.104	0.331
*Size*	5814	22.11	1.204	18.96	27.55	21.27	21.97	22.79
*ROA*	5814	0.0392	0.0724	-1.241	0.480	0.0158	0.0405	0.0699
*Lev*	5814	0.391	0.184	0.0271	0.976	0.243	0.386	0.528
*Fix*	5814	0.200	0.134	0.00103	0.806	0.0975	0.174	0.270
*Growth*	5814	0.191	0.595	-0.862	17.32	-0.00371	0.115	0.266
*Top1*	5814	31.69	13.77	3.622	85.04	21.07	29.95	40.84
*Age*	5814	2.840	0.321	1.609	3.664	2.639	2.890	3.091
*Wage*	5814	11.59	0.412	9.002	14.38	11.32	11.55	11.84
*HHI*	5814	0.0112	0.0313	0.000115	0.358	0.00140	0.00319	0.00834
*PGDP*	5814	11.26	0.403	10.17	12.01	10.97	11.30	11.55

*Notes*: In the table, p25, p50, and p75 represent the 25%, 50%, and 75% quantiles of the sample respectively.

*Source*: China Economic and Financial Research Database (CSMAR) and China Statistical Yearbook.

## 4. Empirical results

### 4.1. The main regression results

**Fig 5:**
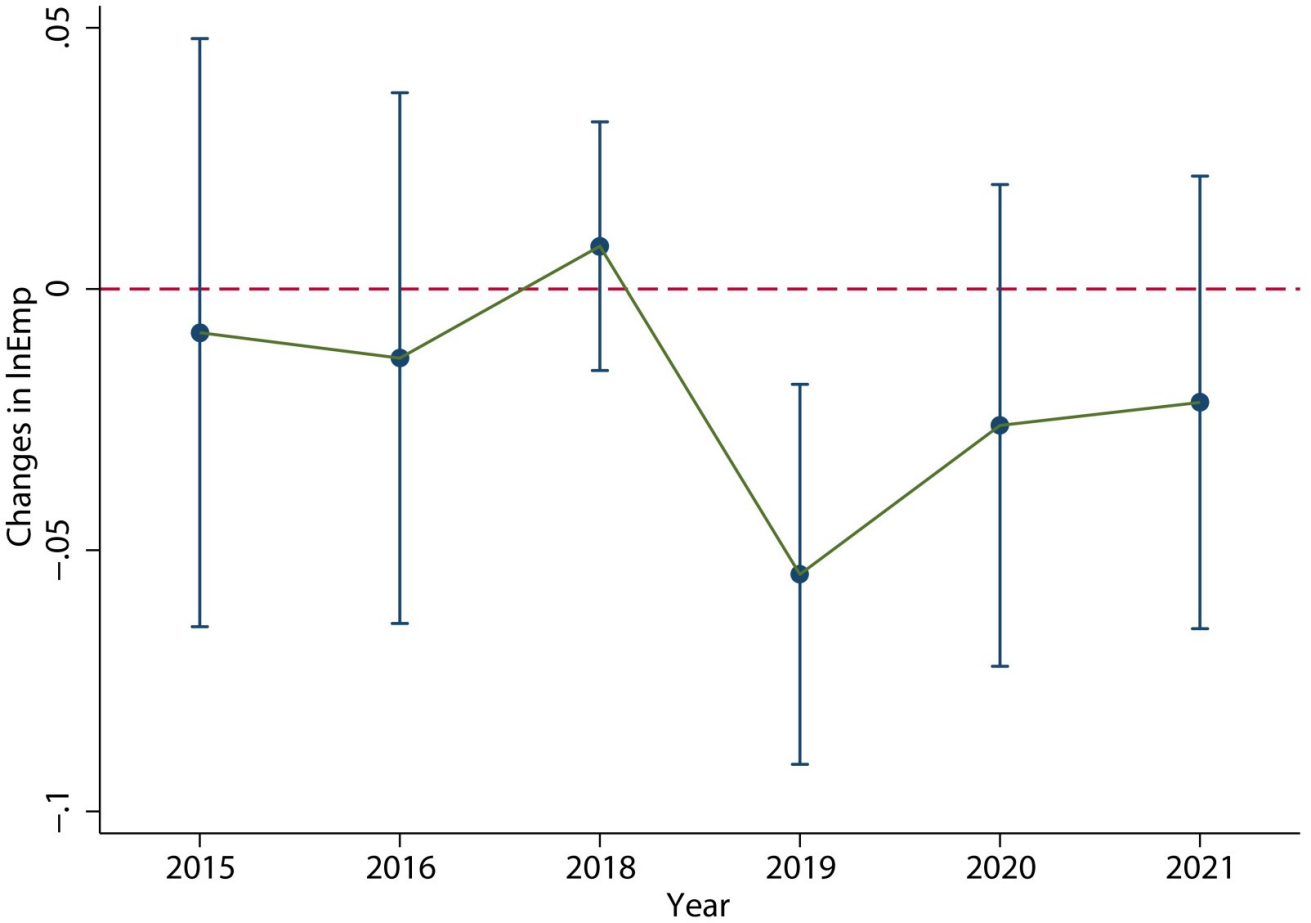
The main regression results. It shows the values of coefficient *β*_*k*_ and its 90% confidence interval through 2017 to 2021. It can be seen that after the implementation of the policy, there is an obvious *V-shaped fluctuation trend*. This figure was generated in STATA17.

Fig 5 shows the estimated coefficient *β*_*k*_ and its 90% confidence interval through 2017 to 2021. The coefficient *β*_*k*_ represents the difference in the scale of labor employment of enterprises with different intensity of VAT credit refunds in the year of *k*. Firstly, there was no significant difference in labor employment among enterprises with different intensities of VAT credit refunds in the three years before the policy implementation, i.e., from 2015 to 2017. This is the prerequisite to evaluate the employment effect of the policy based on the benchmark year of 2017. Then, it can be seen that after the implementation of the policy, there is an obvious *V-shaped fluctuation trend*: In 2018, that is the initial stage of the implementation of the VAT credit refunds policy, the average number of laborers employed by the enterprises increased slightly (without decreases), which means that the number of employees in the enterprises did not decreased immediately after the implementation of the policy and the Inference 1 in the theoretical analysis is confirmed. In 2019, which is the follow-up stage of the implementation of VAT credit refunds policy, the average number of laborers employed by the enterprises decreased significantly; and then from 2020 to 2021, the average number of laborers employed by the enterprises increased significantly, which means the decreased employment had been recover. These are in line with Inference 2 in the theoretical analysis.

The detail results are shown in column (1) of Table 4, which clearly shows a significant decrease of enterprises employment in 2019 and a significant recovery from 2020 to 2021, supporting the “*V-shaped fluctuation trend*”. In terms of control variables, *Size*, *ROA*, *Fix* and *Age* are significantly positively correlated with the scale of labor employment, while *Wage* is significantly negatively correlated with the scale of labor employment.

**Table 4:** The results of event study.

Variables	(1) Main regression	(2) Change the measurement of *Emp*	(3) Change intensity measurement	(4) Rule out accelerating depreciation industries	(5) Controling the E&A deducting policy
	ln*Emp*	*ΔEmp*	ln*Emp*	ln*Emp*	ln*Emp*
*Tax*_*refunds* × I_2015_	-0.0084 (0.0342)	0.0046 (0.0234)	-0.0124 (0.0269)	-0.0161 (0.0335)	-0.0132 (0.0353)
*Tax*_*refunds* × I_2016_	-0.0132 (0.0309)	-0.0027 (0.0215)	-0.0060 (0.0224)	-0.0059 (0.0260)	-0.0041 (0.0296)
*Tax*_*refunds* × I_2018_	0.0082 (0.0145)	0.0161 (0.0157)	0.0084 (0.0181)	-0.0096 (0.0230)	0.0070 (0.0145)
*Tax*_*refunds* × I_2019_	-0.0546** (0.0221)	-0.0414* (0.0222)	-0.0471* (0.0266)	-0.0751** (0.0315)	-0.0509** (0.0217)
*Tax*_*refunds* × I_2020_	-0.0261 (0.0280)	-0.0290 (0.0234)	-0.0060 (0.0359)	-0.0448 (0.0439)	-0.0313 (0.0280)
*Tax*_*refunds* × I_2021_	-0.0217 (0.0263)	-0.0020 (0.0277)	-0.0094 (0.0323)	-0.0399 (0.0407)	-0.0254 (0.0267)
L.*Size*	0.4538*** (0.0222)	0.3743*** (0.0393)	0.4692*** (0.0223)	0.4393*** (0.0280)	0.4400*** (0.0224)
*L*.*ROA*	0.1747** (0.0782)	0.0335 (0.0870)	0.2789*** (0.0800)	0.1814** (0.0888)	0.1930** (0.0751)
*L*.*Lev*	0.0398 (0.0655)	0.0542 (0.1097)	0.1392** (0.0670)	0.0833 (0.0776)	0.0624 (0.0641)
*L*.*Fix*	0.2567** (0.1023)	0.2896*** (0.1116)	0.1737* (0.1026)	0.3007** (0.1267)	0.2558** (0.1028)
*L*.*Growth*	0.0127 (0.0125)	0.0415 (0.0260)	0.0022 (0.0077)	0.0048 (0.0081)	0.0145 (0.0135)
*L*.*Top1*	0.0014 (0.0014)	0.0012 (0.0027)	0.0016 (0.0013)	0.0024 (0.0016)	0.0014 (0.0014)
*L*.*Age*	0.5636*** (0.1347)	0.4720** (0.1847)	0.4476*** (0.1241)	0.4073*** (0.1297)	0.5709*** (0.1347)
*L*.*Wage*	-0.2330*** (0.0275)	-0.1673*** (0.0424)	-0.2753*** (0.0296)	-0.2526*** (0.0353)	-0.2280*** (0.0281)
*L*.*HHI*	-0.4111 (0.5536)	-0.7848 (0.7237)	-0.3848 (0.4544)	-0.0477 (0.4867)	-0.3529 (0.5595)
*L*.*PGDP*	-0.0986 (0.0807)	-0.0100 (0.0817)	-0.0524 (0.0833)	-0.0594 (0.1253)	-0.0875 (0.0805)
*Constant*	-0.0031 (1.0549)	-6.7643*** (1.3504)	-0.1129 (1.0524)	0.3889 (1.4853)	0.0820 (1.0667)
Time-fixed effect	Yes	Yes	Yes	Yes	Yes
Individual-fixed effect	Yes	Yes	Yes	Yes	Yes
N	5814	5814	5881	3831	5739
Adj R^2^	0.3691	0.1935	0.4076	0.4198	0.3778

Standard errors in parentheses.

Significance levels:

*** *p* < 0.01,

** *p* < 0.05,

* *p* < 0.1

*Source*: China Economic and Financial Research Database (CSMAR) and China Statistical Yearbook.

### 4.2. Robustness test

#### 4.2.1. Placebo test

**Fig 6:**
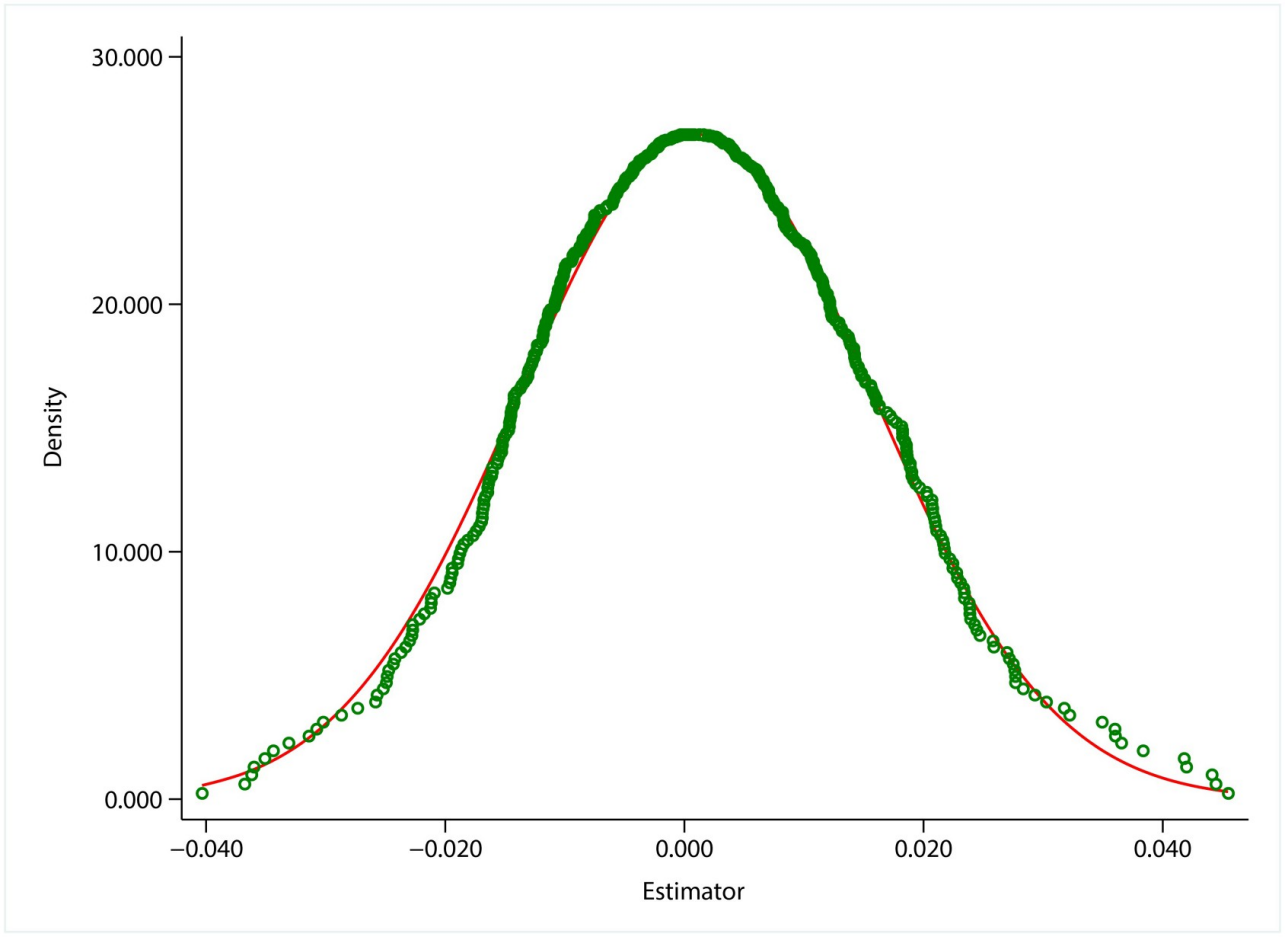
The results of placebo test. The coefficient estimates are all distributed around 0, indicating that random unobserved factors have a small impact on the estimated results. This figure was generated in STATA17.

The results of this paper may be affected by other unobservable factors. To test the degree of interference from random factors, referring to the practice of Ferrara et al. [51] and Liu & Lu [52], a false experiment is constructed by randomly “allocating” the intensity of VAT credit refunds among sample enterprises. The regression is then run and the process is repeated 500 times to plot the distribution of coefficient estimates. The results are shown in Fig 6, and the coefficient estimates are all distributed around 0, indicating that random unobserved factors have a small impact on the estimated results, and the impact of other policies is excluded to a certain extent [53].

#### 4.2.2. Change explained variable measurement

In the main regression analysis, we use the natural logarithm of the number of employees at the end of the year to represent the scale of labor demand. Subsequently, we employ the ratio of the number of employees to total assets in 2017 (recorded as *ΔEmp*) to represent the scale of labor demand, aiming to test the robustness of the main regression results.

**Fig 7:**
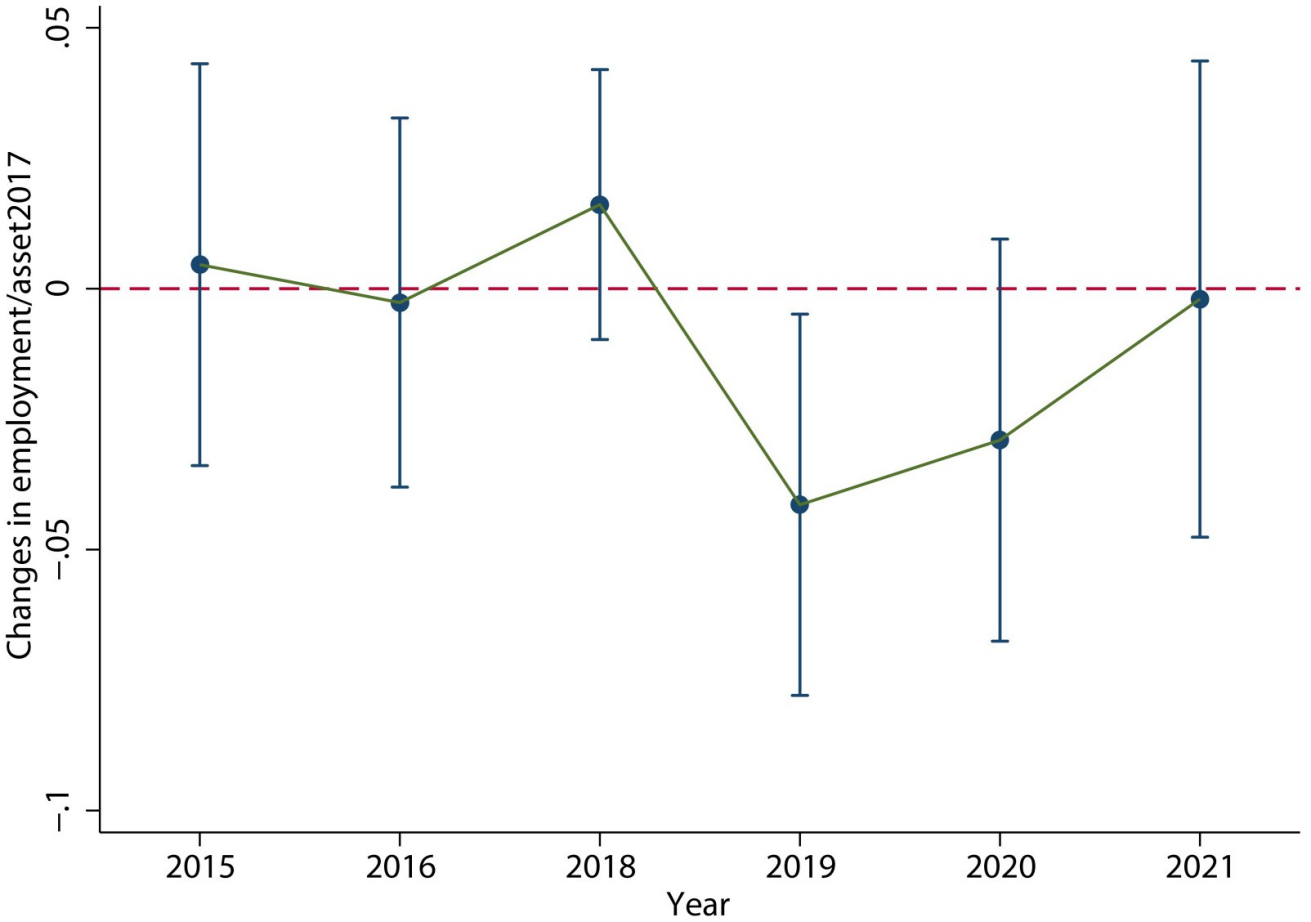
The results of changing the explained variable measurement. When we change the explained variable measurement, the dynamic effect test result is still significant and the *V-shaped fluctuation trend* does not change. This figure was generated in STATA17.

As shown in Fig 7, the results of the dynamic effects test are robust, and there is not much change in the *V-shaped fluctuation trend*. The specific regression results are shown in column (2) of Table 4, which clearly shows a significant decrease in employment in 2019. This is consistent with the conclusion of the main regression.

#### 4.2.3. Change intensity measurement

**Fig 8:**
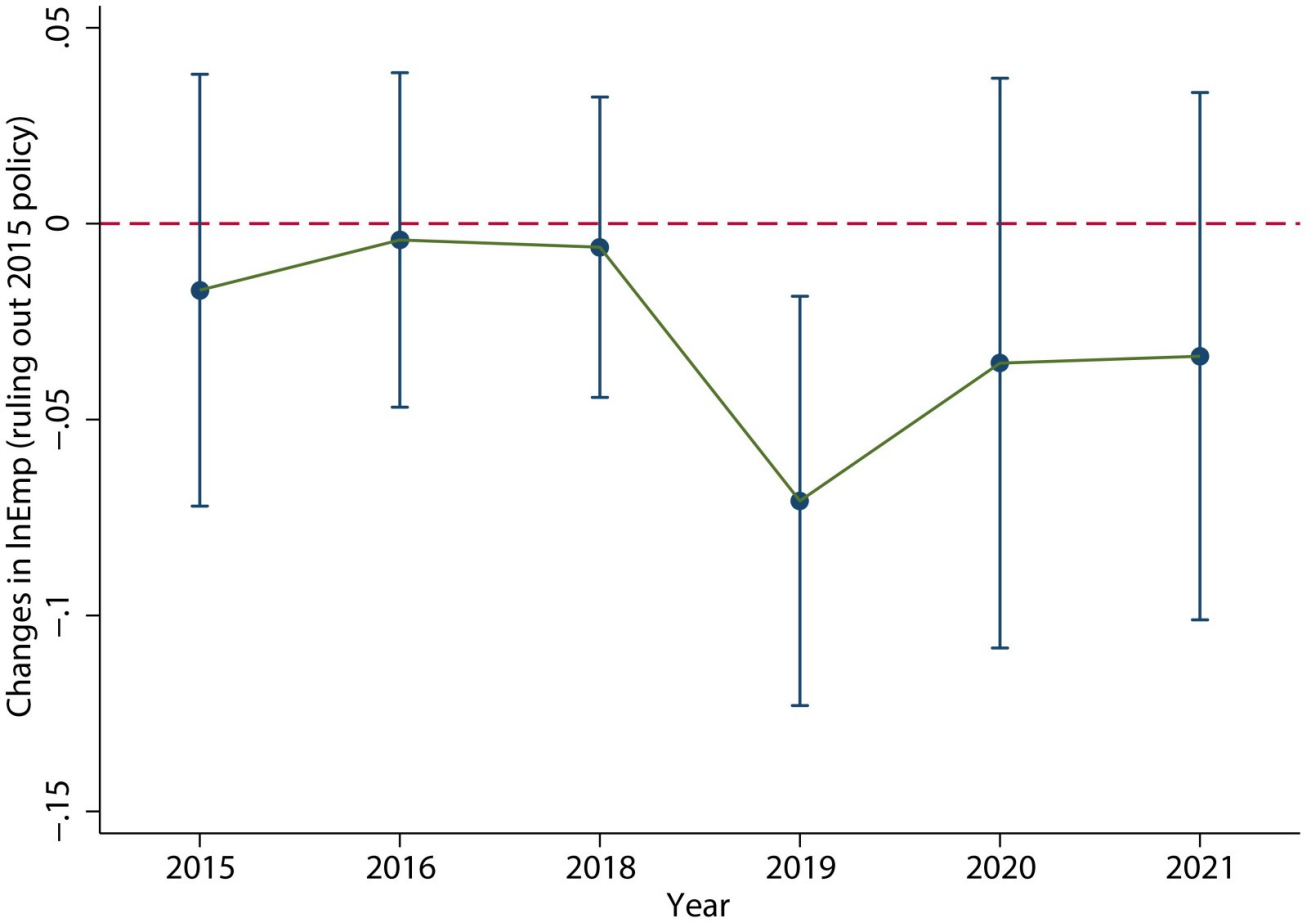
The results of changing the intensity measurement. When we change the intensity measurement, the dynamic effect test result is still significant and the *V-shaped fluctuation trend* does not change. This figure was generated in STATA17.

In the main regression, we used the VAT credit refunds intensity obtained by fitting the past 6 quarterly data. Possibly, the results would be better if more periods of data were used for fitting. Therefore, we used the past 10 quarterly data to fit and obtain a new VAT credit refunds intensity. As shown in Fig 8, the results of the dynamic effect test are robust, and there is not much change in the *V-shaped fluctuation trend*. The specific regression results are shown in column (3) of Table 4, which clearly shows a significant decrease in employment in 2019. This is consistent with the conclusion of the main regression.

#### 4.2.4. Rule out other explanations

The accelerated depreciation policy of fixed assets in 2015, and the policy on equipment and appliances deduction of enterprise income tax were implemented at the same time as the VAT credit refunds policy in 2018 (Equipment and appliances newly purchased by enterprises during the period from January 1, 2018, to December 31, 2020, with a unit value not exceeding RMB 5 million yuan, are allowed to be included in the current cost in a lump sum and deducted in the calculation of taxable income, and depreciation will not be calculated annually) may have impacts on the evaluation of the tax refunds policy and further verification is needed.

**Fig 9:**
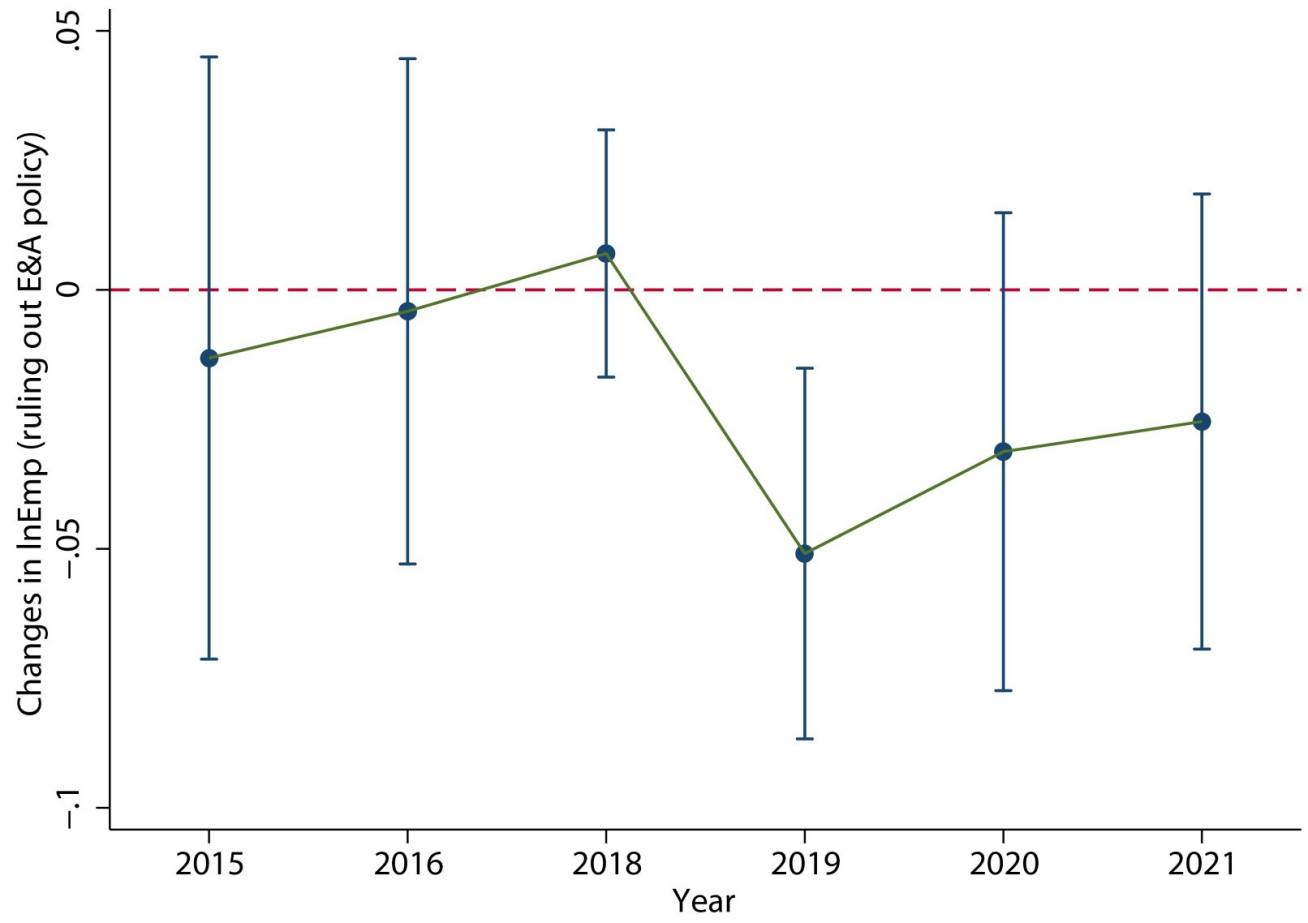
The results of ruling out accelerating depreciation industries in 2015. When ruling out the effect of accelerated depreciation policy of fixed assets, the dynamic effect test result is still significant and the *V*-type trends does not change. This figure was generated in STATA17.

**Fig 10:**
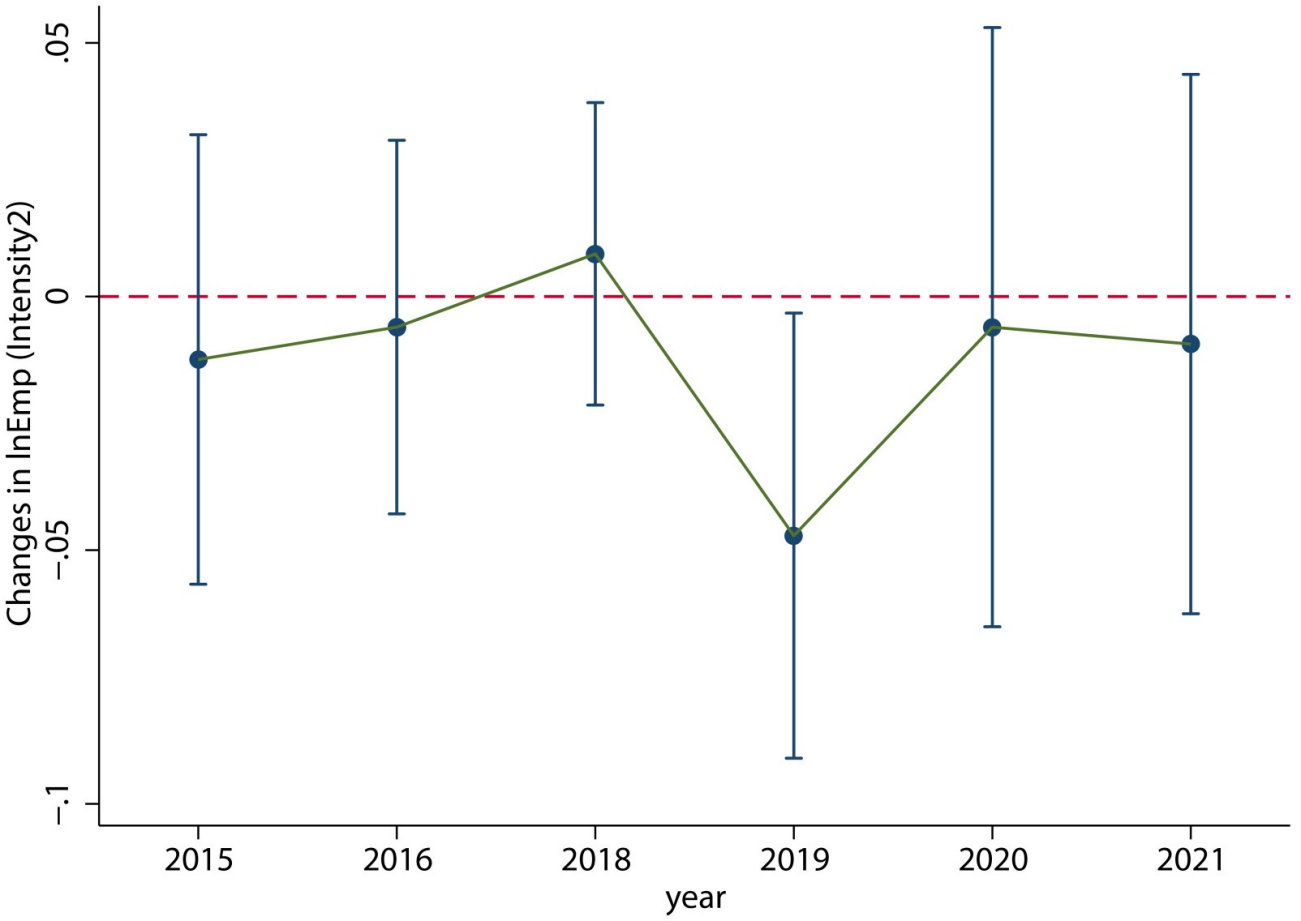
The results of controlling the equipment and appliances deducting policy. When ruling out the effect of the deduction policy for equipment and appliances, the dynamic effect test result is still significant and the *V-shaped fluctuation trend* does not change. This figure was generated in STATA17.

1. Accelerated depreciation policy of fixed assets. We delete the industries that implemented the accelerated depreciation policy of fixed assets in 2015, and then test the effect of the VAT credit refunds policy for the remaining industries. As shown in Fig 9, the dynamic effect test result is still significant and the *V-shaped fluctuation trend* does not change. The specific regression results are shown in column (4) of Table 4, which clearly shows a significant decrease in employment in 2019.
2. Deduction policy for equipment and appliances. The added value of the proportion of the balance of equipment and appliances (balance of fixed assets minus balance of housing and buildings) in the total assets in 2018 compared with that in 2017 is taken as the proxy variable of the deduction policy of equipment and appliances, and the cross term of the proxy variable and *Post* is added to Eq (16) for dynamic test. The result is shown in Fig 10. The *V-shaped fluctuation trend* of the effect of the tax refunds policy is not greatly affected. The specific regression results are shown in column (5) of Table 4, which clearly shows a significant decrease in employment in 2019.

#### 4.2.5. Omitted variable test

The employment effect of VAT credit refunds in 2019 was significantly negative. To make this conclusion more robust, we refer to the methods of Altonji et al. [54] and Oster [55] to test coefficient stability. Altonji et al. [54] indirectly estimate the degree to which the model bias is caused by omitted variables by establishing two regression equations. First, a model containing a limited number of constrained control variables is constructed, and the parameter estimate *β*^*R*^ of the core explanatory variables is obtained through regression. Then, all observable variables are introduced as control variables into the model, and the parameter estimate *β*^*F*^ of the core explanatory variables is obtained through regression. Finally, the omitted variable bias coefficient is calculated according to the formula *σ* = |*β*^*F*^/(*β*^*R*^ − *β*^*F*^)|. The larger the value of *σ*, the smaller the impact of omitted variables on the model regression results. Typically, if *σ* is greater than 1, it indicates that the impact of omitted variables is relatively small. Following this approach, we establish a constrained model and a full model in this paper. The constrained model only introduces core explanatory variables, industry dummy variables, and year dummy variables. The full model adds control variables based on the constrained model. The calculated omitted variable bias coefficient *σ* is greater than 1 (2.7752), indicating that the possibility of bias in our estimation results due to omitted variables is low.

Oster [55] argues that when there may be omitted variables in the model, a consistent estimate of the true parameters can be obtained based on β* = β*(*R*_max_, *δ*). Among them, *δ* represents the selection ratio. *R*_max_ represents the maximum goodness of fit of the model regression when controlling for omitted variables. There are two main strategies for testing the impact of omitted variables: (1) It is to let take *δ* a value of 1 and take *R*_max_ a value of 1.3 times the current regression goodness of fit. If falls within the 95% confidence interval of the estimated coefficient, it indicates that the omitted variable problem is not severe. (2) It is to let take *R*_max_ a value of 1.3 times the current regression goodness of fit, β* = 0, calculate the value of |*δ*|, and if |*δ*| > 1 it indicates that the impact of omitted variables on coefficient estimation is not significant. The test results in Table 5 show that the impact of omitted variables on the model estimation results in this paper is relatively small.

**Table 5:** The results of the omitted variable test using Oster’s (2019) method.

Testing strategy	Criteria	Actual result	Pass or not
(1)	β* = β*(*R*_max_, *δ*) ∈ [-0.097977, -0.011265]	-0.09752	Pass
(2)	|*δ*| > 1	-3.934626	Pass

## 5. Further test

### 5.1. Heterogeneity analysis

According to Chen and Chen [30], the capital-labor elasticity of substitution in each sub-industry of Manufacturing in China is shown in Table 6. The greater the elasticity of substitution is, the more difficult it is for labor demand to increase when the price of capital falls. According to the median of the elasticity values of the industries in which the enterprises in the sample are located, C27, C33, C35, C36, C37 and C39 with larger elasticity of substitution are divided into one group (the group with large elasticity of substitution, including 469 out of 945 enterprises), and other industries are divided into one group (the group with small elasticity of substitution, including the remaining 476 out of the 945 enterprises).

**Table 6:** Predicted value of cross-elasticity of labor demand to capital cost in the pilot industry.

Industry	Industry code	*σ*	Industry	Industry code	*σ*
Manufacture of Chemical Raw Material and Chemical Products	C26	2.11	**Manufacture of Special Purpose Machinery**	**C35**	**2.15**
**Manufacture of** **Medicines**	**C27**	**2.81**	**Manufacture of Transport Equipment**	**C36, C37**	**2.14**
Manufacture of Chemical Fibres	C28	1.31	Manufacture of Electrical Machinery and Apparatus	C38	1.97
Manufacture of Non-metallic Mineral Products	C30	2.08	**Manufacture of Computers, Communication and Other Electronic Equipment**	**C39**	**3.98**
Manufacture of Metal Products	**C33**	**2.14**	Manufacture of Measuring Instruments and Machinery	C40	2.02
Manufacture of General Purpose Machinery	C34	2.03			

*Source*: Manually collated based on the research of Chen and Chen [30].

**Fig 11:**
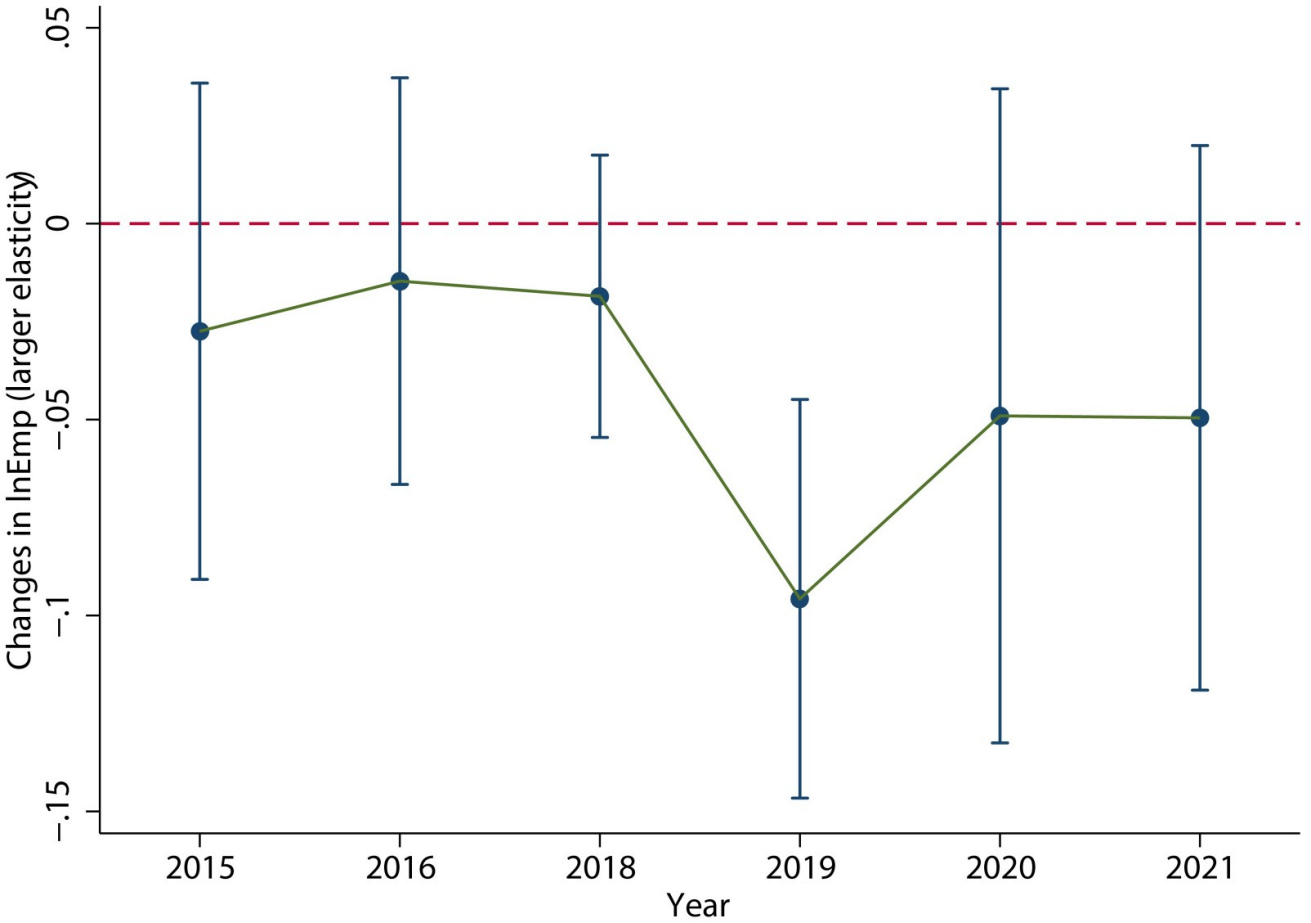
Dynamic effects of C27, C33, C35, C36, C37 and C39 in the industry. The labor demand of the group with large elasticity of substitution decreased significantly in 2019. This figure was generated in STATA17.

As shown in Fig 11, the labor demand of the group with large elasticity of substitution did not increase in 2018, but decreased significantly in 2019. The specific regression results are shown in column (1) of Table 7, which clearly shows a significant decrease in employment in 2019.

**Table 7:** The results of heterogeneity analysis.

Variables	(1) Group with large elasticity of substitution	(2) Group with small elasticity of substitution
	ln*Emp*	ln*Emp*
*Tax*_*refunds* × I_2015_	-0.0275 (0.0384)	0.0340 (0.0356)
*Tax*_*refunds* × I_2016_	-0.0147 (0.0315)	-0.0066 (0.0281)
*Tax*_*refunds* × I_2018_	-0.0185 (0.0219)	0.0487 (0.0300)
*Tax*_*refunds* × I_2019_	-0.0957*** (0.0309)	0.0045 (0.0315)
*Tax*_*refunds* × I_2020_	-0.0491 (0.0506)	0.0173 (0.0306)
*Tax*_*refunds* × I_2021_	-0.0496 (0.0422)	0.0326 (0.0382)
Control Variables	Yes	Yes
Time-fixed effect	Yes	Yes
Individual-fixed effect	Yes	Yes
N	3153	3235
Adj R^2^	0.4070	0.4105

Standard errors in parentheses.

Significance levels:

*** *p* < 0.01,

** *p* < 0.05,

* *p* < 0.1

*Source*: China Economic and Financial Research Database (CSMAR) and China Statistical Yearbook.

**Fig 12:**
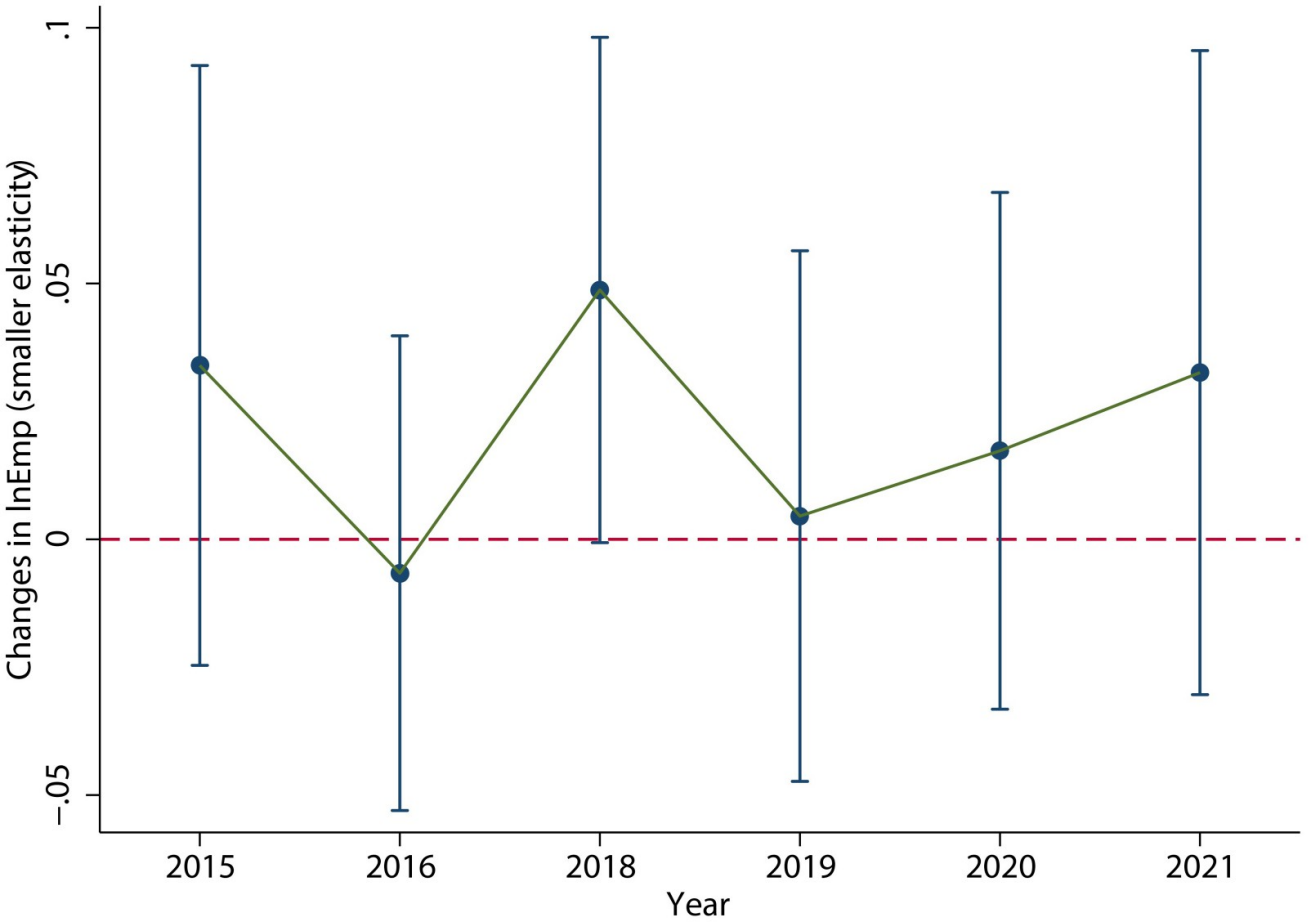
Dynamic effects of other industries of manufacture (except C27, C33, C35, C36, C37 and C39). The labor demand of the group with small elasticity of substitution did not decrease significantly in 2019. This figure was generated in STATA17.

In contrast, the labor demand of the group with small elasticity of substitution (Fig 12) increased significantly in 2018, but did not decrease significantly in 2019. The specific regression results are shown in column (2) of Table 7, which clearly shows a significant increase in employment in 2018. This is consistent with the conclusions of the model analysis.

### 5.2. Mechanism analysis

According to the above model analysis, after the implementation of the policy, the output and capital of enterprises will steadily increase, although the labor demand will show a *V-shaped fluctuation trend*. To verify this, a dynamic effect test is designed.

(1) To examine the impact of the intensity of tax credit refunds on firms’ operating profits, the following model is used:

$$OP_{i,t}=\alpha +\sum_{k=2015,k\neq 2017,}^{2021}\beta_{k}\left[Tax\_refunds_{i}\times \text{I}(k=t\;)\right]\;+\gamma X_{i,t-1}+u_{i}+\lambda_{t}+\varepsilon_{it}$$ (16)

Where *OP* represents the operating profit per unit of assets for the current year. The remaining variables are consistent with Eq (15).

**Fig 13:**
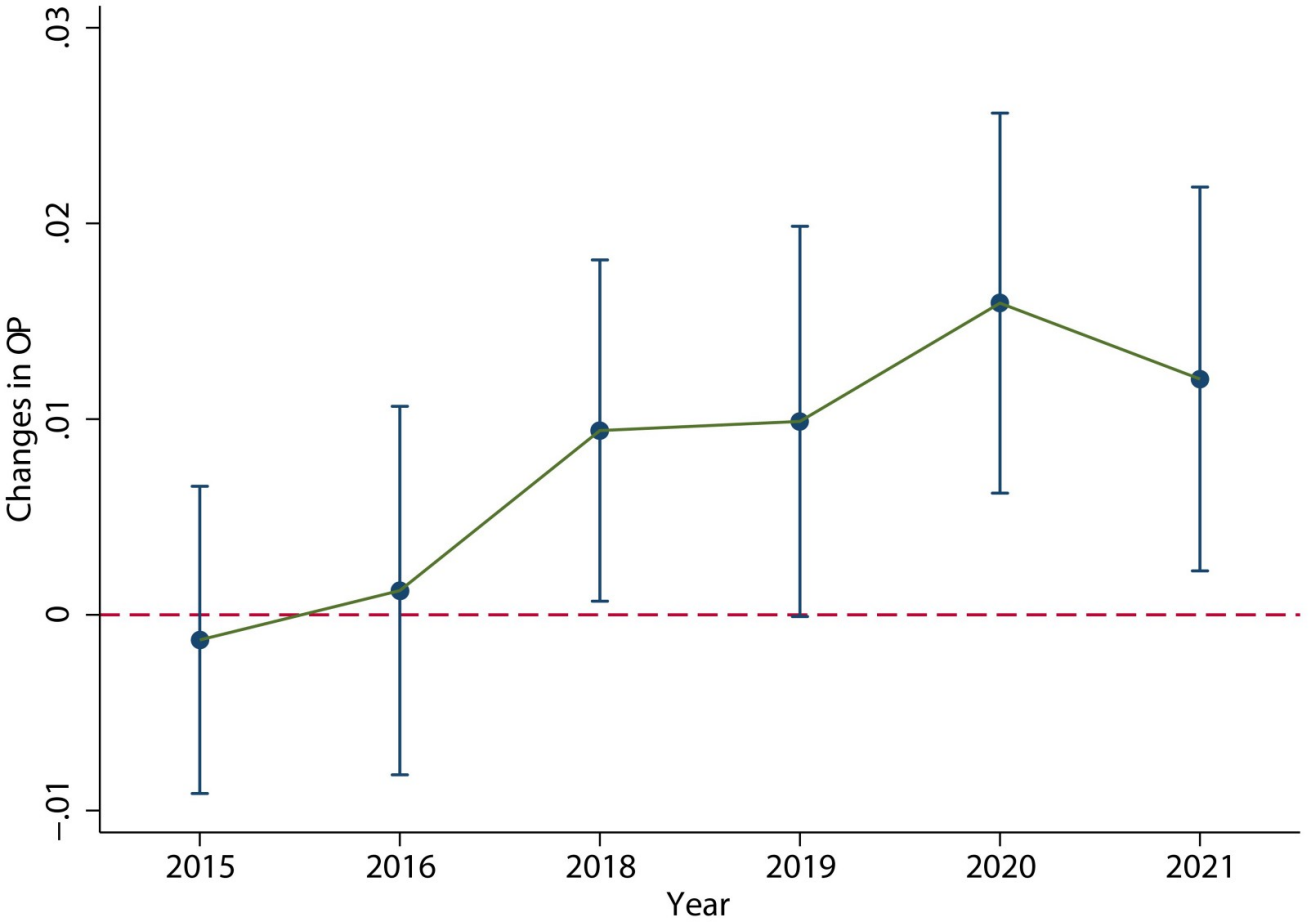
Dynamic effects of business profit. After the implementation of the policy, the operating profits per unit of assets have steadily increased since 2018. This figure was generated in STATA17.

Fig 13 shows the dynamic effects of VAT credit refunds on enterprise operating profits. It can be seen that after the implementation of the policy, the operating profits per unit of assets of the enterprises have steadily increased since 2018.

Furthermore, we use a mediation effect model, which is composed of Eqs (15), (16), and (17), to examine the mechanism through which VAT credit refunds affect enterprises’ operating profits and subsequently employment.


$$\begin{array}{ll} \text{ln}\;Emp_{i,t} & =\alpha +\sum_{k=2015,k\neq 2017}^{2021}\beta_{k}\left[Tax\_refunds_{i}\times \text{I}(k=t)\right]\;+\;\phi OP_{i,t}\\ \; & +\gamma X_{i,t-1}+u_{i}+\lambda_{t}+\varepsilon_{it} \end{array}$$ (17)

The results, as shown in column (2) of Table 8, indicate that the coefficient *ϕ* is significant at 1% significance level.

**Table 8:** The results of the mediation effect model analysis.

Variables	(1)	(2)	(3)	(4)
	*OP*	ln*Emp*	ln*K*	ln*Emp*
*Tax*_*refunds* × I_2015_	-0.0013 (0.0048)	-0.0058 (0.0262)	-0.0409 (0.0328)	0.0027 (0.0257)
*Tax*_*refunds* × I_2016_	0.0012 (0.0057)	-0.0140 (0.0219)	0.0206 (0.0322)	-0.0181 (0.0220)
*Tax*_*refunds* × I_2018_	0.0094* (0.0053)	0.0044 (0.0193)	0.0715** (0.0321)	-0.0080 (0.0187)
*Tax*_*refunds* × I_2019_	0.0099 (0.0061)	-0.0604** (0.0259)	0.0651* (0.0350)	-0.0714*** (0.0248)
*Tax*_*refunds* × I_2020_	0.0159*** (0.0059)	-0.0347 (0.0348)	0.0814** (0.0409)	-0.0472 (0.0315)
*Tax*_*refunds* × I_2021_	0.0121** (0.0060)	-0.0264 (0.0329)	0.0922** (0.0440)	-0.0425 (0.0305)
*OP*		0.3240*** (0.0618)		
ln*K*				0.2170*** (0.0213)
Control Variables	Yes	Yes	Yes	Yes
Time-fixed effect	Yes	Yes	Yes	Yes
Individual-fixed effect	Yes	Yes	Yes	Yes
N	6331	6331	6331	6331
Adj R^2^	0.0738	0.4171	0.5524	0.4712

Standard errors in parentheses.

Significance levels:

*** *p* < 0.01,

** *p* < 0.05,

* *p* < 0.1.

*Source*: China Economic and Financial Research Database (CSMAR) and China Statistical Yearbook.

From the regression results of Eq (15), it can conclude that the VAT credit refunds policy has a significant dynamic impact on employment. According to the regression results of Eq (16), it is evident that the implementation of the VAT credit refunds policy significantly promotes the operating profits of the enterprises. Furthermore, the positive and significant coefficient of corporate operating profits in Eq (17) indicates that corporate operating profits continuously promote employment, and the consistent increase in corporate operating profits ensures the recovery of employment after downward fluctuations.

(2) To examine the impact of the intensity of VAT credit refunds on enterprises’ fixed capital, the following model is used:

$$\text{ln}\;K_{i,t}=\alpha +\sum_{k=2015,k\neq 2017}^{2021}\beta_{k}\left[Tax\_refunds_{i}\times \text{I}(k=t)\right]\;+\gamma X_{i,t-1}+u_{i}+\lambda_{t}+\varepsilon_{it}$$ (18)

Where *K* represents the balance of fixed assets excluding buildings and structures at the end of the year for the enterprises. The remaining variables are consistent with Eq (15).

**Fig 14:**
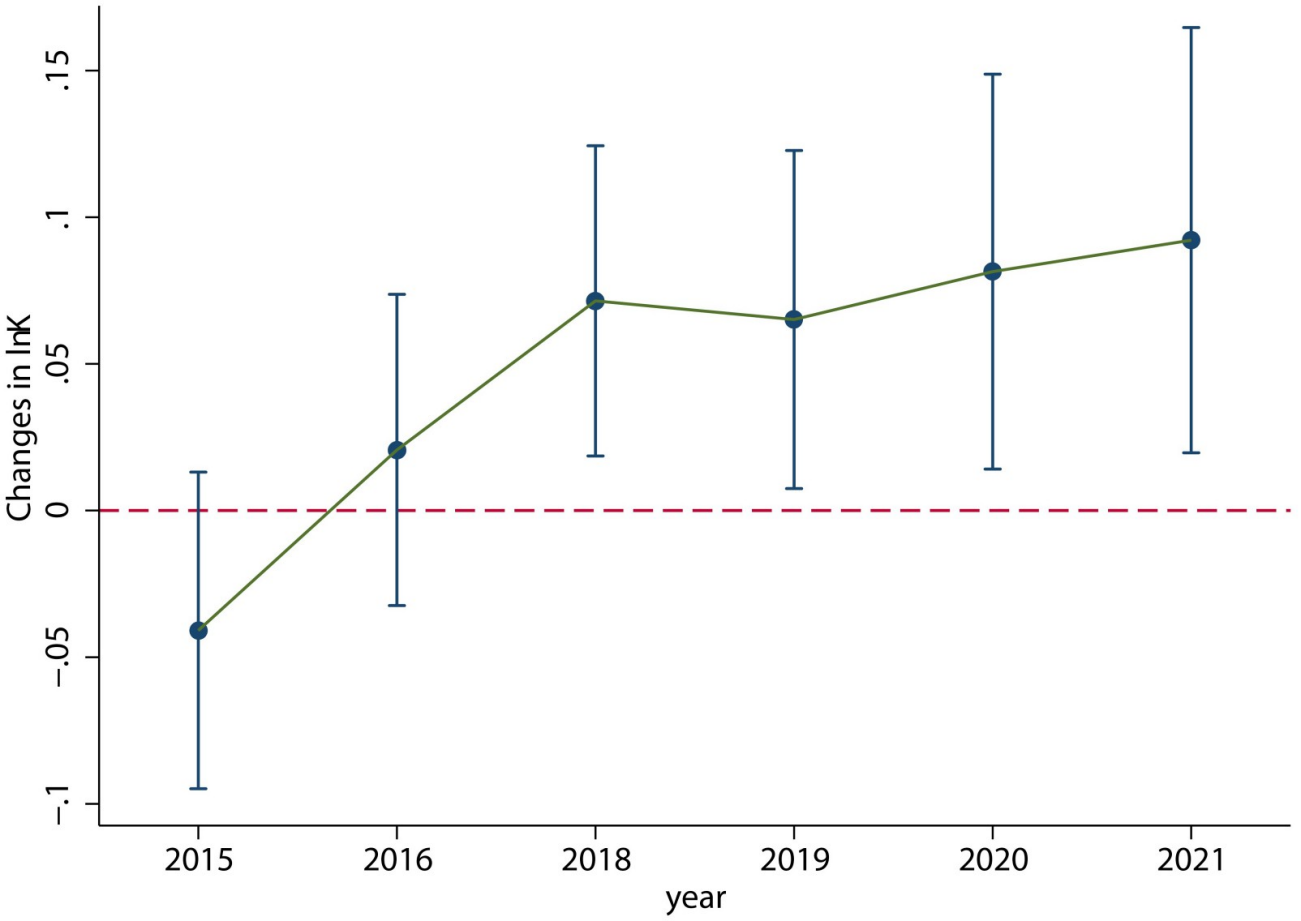
Dynamic effects of VAT credit refunds on the balance of fixed assets minus the balance of houses and buildings. The capital employed by enterprises increased significantly after 2018. This figure was generated in STATA17.

Fig 14 shows the dynamic effects of VAT credit refunds on the fixed capital of enterprises, using the balance of fixed assets minus the balance of houses and buildings, and it can be seen that the capital employed by enterprises increased significantly after 2018. Due to the substitution effect of capital on labor, the demand for labor decreased in 2019 following capital increase.

Furthermore, we use a mediation effect model, which is composed of Eqs (15), (18) and (19), to examine the mechanism through which VAT credit refunds affect enterprises’ fixed assets and subsequently employment.


$$\begin{array}{ll} \text{ln}\;Emp_{i,t} & =\alpha +\sum_{k=2015,k\neq 2017}^{2021}\beta_{k}\left[Tax\_refunds_{i}\times \text{I}(k=t)\right]\;+\;\phi \text{ln}\;K_{i,t}\\ \; & +\gamma X_{i,t-1}+u_{i}+\lambda_{t}+\varepsilon_{it} \end{array}$$ (19)

The results, as shown in column (4) of Table 8, indicate that the coefficient *φ* is significant at 1% significance level.

From the regression results of Eq (15), it can conclude that the VAT credit refunds policy has a significant dynamic impact on employment. According to the regression results of Eq (18), it is evident that the fixed capital of enterprises was increased immediately and significantly after the implementation of the VAT credit refunds policy. Furthermore, the significant coefficient of corporate operating profits in Eq (19) indicates that average effects of the increased fixed capital of enterprises on the employment is positive, and the consistent increase in the increased fixed capital of enterprises also ensures the recovery of employment after downward fluctuations.

Based on the above mechanism analysis, the policy effects on “operating profits” and “fixed capital” precisely validate how the *V-shaped fluctuation trend* of the employment is formed. On the one hand, after the implementation of the policy, the relative decline in capital prices increases the demand for capital, and the capital-labor substitution elasticity being less than 1 results in capital substituting for labor, causing employment to change from no significant difference before the policy implementation to significantly negative. This is the “\” part of the *V-shaped fluctuation trend*. On the other hand, due to the continuous increase in output, as predicted by the theoretical analysis, the increase in output leads to an increase in the demand for both capital and labor, thus enabling the labor demand of enterprises to rebound to a state of no significant difference after a short period of decline. This is the “/” part of the *V-shaped fluctuation trend*.

## 6. Conclusion and policy implications

Tax incentive policies provide support for enterprises and promote their development, but usually have an impact on labor demand by causing the price of capital to fall. Previous studies have recognized the substitution effect caused by the decrease in capital prices, and also found the scale effect brought by the expansion of production scale. However, the real effect can only be tested empirically. Due to the lack of support from mathematical analysis, the results of empirical tests will be contradictory for different samples and period (some believe that it promotes employment, while others believe that it inhibits employment), which weakens the scientific nature of policy evaluation.

This paper establishes a mathematical model to analyze the impact of VAT credit refunds on enterprise labor demand, and finds that it presents a *V-shaped fluctuation trend*, which is different from the conclusion of “either rise or fall” in previous studies. In the initial implementation of the policy, due to the existence of layoff costs, the cost line of enterprises bends, and enterprises with capital-labor substitution elasticity greater than 1 usually will not reduce the employment of labor, because they have completed the labor configuration before the implementation of the policy. When enterprises enter the next production cycle where labor can be freely allocated, the labor employment of enterprises with capital-labor elasticity of substitution greater than 1 will decline compared to those without the policy. In the long run, with the increase of output effect, labor demand will recover. The overall evaluation will be affected by the choice of period after the implementation of the policy.

In the ex-ante analysis, we estimate the impact of the policy on Chinese enterprises labor demand through parameters calibration. Then, using the data of China’s A-share listed enterprises for empirical testing, the results are consistent with that of ex-ante analysis. In addition, the heterogeneity analysis finds that the greater the capital-labor elasticity of substitution is, the more severe the degree of the *V-shaped fluctuation* is. After the implementation of the policy, the output and capital stock of the enterprise increases, which verifies the relevant transmission mechanism. This study provides a more detailed perspective for fully understanding how the policy of VAT credit refunds policy affects employment, and contributes to the scientific evaluation of the policy effect. Based on this, this study provides useful methods for policymakers to effectively evaluate the employment effect of similar tax incentive policies before their implementation and conduct post-event testing after the implementation of the policies.

The main limitation of this study is the relatively limited empirical sample. The data used in this study are only a part of Chinese enterprises, which are listed companies. If more enterprise data can be obtained, the results of empirical analysis will be more comprehensive and representative.

The research in this paper has strong theoretical and practical significance. In terms of theory, this paper provides a perfect model derivation, parameter calibration, ex-ante analysis and ex-post empirical test for a more scientific evaluation of how tax incentive policies affect employment. In terms of practices, it provides the following implications for policymakers: Firstly, it is necessary to further improve preferential tax policies such as VAT credit refunds, as these policies can indeed help enterprises improve their business conditions. The scope of application of these policies should be adjusted and expanded based on different policy objectives to fully leverage the role of these tax incentive policies. Secondly, it is crucial to continuously enhance forward-looking research on policy effects. Emphasis should be placed on pre-policy investigation and research, conducting thorough pre-analysis of the target groups of these policies, and examining the effects of policy implementation from multiple perspectives, especially the impact on employment. Thirdly, for employment fluctuations caused by policies, such as the short-term employment fluctuations resulting from the VAT credit refund policy discussed in this article, corresponding countermeasures should be taken. For instance, increasing employment training to assist workers in making better job transitions and improving employment information platforms to enable job seekers to access recruitment information promptly.

## Supporting information

S1 File (DOCX)

S1 Dataset (ZIP)
