# Supplementary material for: The impact of China’s value-added tax credit refunds on enterprise labor demand: Ex-ante analysis and ex-post test
Source: PLoS One. 2024 Jun 11;19(6):e0305249. doi: 10.1371/journal.pone.0305249 (PMC11166334; doi:10.1371/journal.pone.0305249)
Supplement: S1 File — (DOCX) [file pone.0305249.s001.docx]

The impact of China's value-added tax credit refunds on enterprise labor demand: Ex-ante analysis and ex-post test

**Appendix A**

If the profits of expanding production are greater than putting the funds into bank, then the enterprise will choose to expand production. Now let's compare and analyze the two options.

1. Putting the funds into bank and getting the principal and interest at the end of the period. In this case, at the end of the first stage, the enterprise sells product *Y*_1,1_, pays output tax at the tax rate of *t* without any deduction, but can obtain the principal and interest(1+*r*)$t\bar{R}K_{1,0}^{*}$of the tax refund. Therefore, the total benefit Π_1a_ is:

（A1）

Where$Y_{1,1}^{*}$is the optimal output for the first stage, which equals to that when carrying forward for deduction. However, compared to the case of carrying forward the deduction, the total profits of the enterprise is an additional interest generated from the tax credit. Therefore, the case of having VAT credit refund is more beneficial for the enterprise than that of carrying forward the deduction.

2. To purchase production factors using the tax refund and expand output. For enterprises that have received the tax credit refund, the cost constraint becomes:

（A2）

This is a new iso-cost line. The absolute values of the slopes of this new iso-cost line and the old actual iso-cost line represented by Eq (5) are: , indicating that the actual price of capital used by the enterprise has decreased from the case of carrying forward the deduction, from $[1+rt/(1+r)]\bar{R}$ to$[1\text{-}t/(1+r)]\bar{R}$.

Faced with the new iso-cost line, theoretically, the enterprise can reallocate capital and labor inputs, and the optimal input combination ($K_{1,0}^{**}$,$L_{1,0}^{**}$) is:

（A3）

where .

Whether the enterprise will expand production? That is, it needs to determine how the enterprise chooses between putting funds into the bank and using the funds to purchase production factors. Since the quantity of capital and labor at the new optimal configuration point ($K_{1,0}^{**}$,$L_{1,0}^{**}$) does not increase in the same proportion as the original capital-labor configuration$\text{（}K_{1,0}^{*},L_{1,0}^{*}\text{）}$, it is not easy to calculate the relationship between the new optimal output and. As a compromise, we calculate the output situation when the enterprise allocates capital and labor using the VAT credit refund funds in the same proportion as before. Of course, this output is certainly less than or equal to the optimal output, meaning that the enterprise will produce at least the output corresponding to this point, which is a conservative output point. Assuming that the newly allocated capital and labor of the enterprise are$\Delta K\text{ and }\Delta L$respectively.

Then:

（A5）

Get: , . Due to the constant returns to scale, the newly increased output is 。At the end of the first stage, the total profit of the enterprise Π_1b_ is:

（A6）

Because:

（A7）

While:

（A8）

Before the policy was implemented, the enterprise would only start production if it expects the total profit at the end of the first stage to be greater than 0 at the beginning of the zeroth stage. In other words, the following condition needs to be met:

（A9）

Therefore, when *μ*≥1, Equation (A7) is always greater than 0. Although it has not yet reached the optimal output, it has already demonstrated that expanding production is more profitable than depositing funds in the bank. Considering the actual situation in China, the return to scale of Chinese industrial enterprises is at around 1.2. So, it can be concluded that enterprises will choose to expand production.

**Appendix B**

1. Derivation process of Eq (4).

The enterprise optimization problem is:

(B1)

Constructing the *Lagrange* function:

(B2)

And find the first-order conditions for *L*, *K*, and *λ* respectively:

(B3)

(B4)

(B5)

First, divide Eq (B4) by Eq (B3) to eliminate *λ*, resulting in:

(B6)

Set. From the above equation, we can obtain:

$K_{1,0}=L_{1,0}\left(1+\frac{tr}{1+r}\right)^{-\frac{1}{(1+\rho)}}I$ (B7)

Then, substituting Eq (B7) into Eq (B5), we can find the optimal labor demand$L_{1,0}^{*}$:

(B8)

Finally, substituting the result into Eq (B7), we can find the optimal capital demand$K_{1,0}^{*}$:

(B9)

That is, the optimal combination is:

(B10)

Where .

2. Derivation process of Eq (7).

The actual iso-cost line of the enterprise is , that is:

(B11)

So, the enterprise optimization problem becomes:

(B12)

By constructing the *Lagrange* function to solve the first-order conditions, the remaining solution process is similar to the derivation process of Eq (4).
